# Feasibility of Applied Gaming During Interdisciplinary Rehabilitation for Patients With Complex Chronic Pain and Fatigue Complaints: A Mixed-Methods Study

**DOI:** 10.2196/games.5088

**Published:** 2016-04-01

**Authors:** Miel A P Vugts, Margot C W Joosen, Alfonsus H M M van Bergen, Hubertus J M Vrijhoef

**Affiliations:** ^1^ Tranzo Scientific Center for Care and Welfare Tilburg School of Social and Behavioral Sciences Tilburg University Tilburg Netherlands; ^2^ Ciran Rehabilitation Centers Venlo Netherlands; ^3^ Saw Swee Hock School of Medicine National University of Singapore Singapore Singapore

**Keywords:** behavioral medicine, therapy, computer-assisted, computer games, mind-body therapies, patient acceptance of health care, feasibility studies, fatigue syndrome, chronic, fibromyalgia, musculoskeletal pain

## Abstract

**Background:**

Applied gaming holds potential as a convenient and engaging means for the delivery of behavioral interventions. For developing and evaluating feasible computer-based interventions, policy makers and designers rely on limited knowledge about what causes variation in usage.

**Objective:**

In this study, we looked closely at why and by whom an applied game (LAKA) is demanded and whether it is feasible (with respect to acceptability, demand, practicality, implementation, and efficacy) and devised a complementary intervention during an interdisciplinary rehabilitation program (IRP) for patients with complex chronic pain and fatigue complaints.

**Methods:**

A mixed-methods design was used. Quantitative process analyses and assessments of feasibility were carried out with patients of a Dutch rehabilitation center who received access to LAKA without professional support during a 16-week interdisciplinary outpatient program. The quantitative data included records of routinely collected baseline variables (t0), additional surveys to measure technology acceptance before (t1) and after 8 weeks of access to LAKA (t2), and automatic log files of usage behavior (frequency, length, and progress). Subsequently, semistructured interviews were held with purposively selected patients. Interview codes triangulated and illustrated explanations of usage and supplemented quantitative findings on other feasibility domains.

**Results:**

Of the 410 eligible patients who started an IRP during the study period, 116 patients participated in additional data collections (108 with problematic fatigue and 47 with moderate or severe pain). Qualitative data verified that hedonic motivation was the most important factor for behavioral intentions to use LAKA (*P*<.001). Moreover, quotes illustrated a positive association between usage intentions (t1) and baseline level (t0) coping by active engagement (Spearman *ρ*=0.25; *P*=.008) and why patients who often respond by seeking social support were represented in a group of 71 patients who accessed the game (*P*=.034). The median behavioral intention to use LAKA was moderately positive and declined over time. Twenty patients played the game from start to finish. Behavioral change content was recognized and seen as potentially helpful by interview respondents who exposed themselves to the content of LAKA.

**Conclusions:**

Variation in the demand for applied gaming is generally explained by perceived enjoyment and effort and by individual differences in coping resources. An applied game can be offered as a feasible complementary intervention for more patients with complex chronic pain or fatigue complaints by embedding and delivering in alignment with patient experiences. Feasibility, effectiveness, and cost-effectiveness can be evaluated in a full-scale evaluation. New observations elicit areas of further research on the usage of computer-based interventions.

##  Introduction

### Background

Computer-based interventions (CBIs) can be effective alternatives or complements to face-to-face delivery in psychological treatment and chronic illness management [1-4]. However, systematic reviews on effectiveness of CBIs have concluded that sizable and heterogeneous proportions of patients stop using CBIs before completion [5-7]. Nonusage attrition in CBI studies depends on factors such as therapist involvement, demographics, computer self-efficacy, and health status [6-11]. As a strategy to improve patient engagement, some CBI designs have incorporated interactive features [12,13]. Interactive and visual-enriched designs may support patient demand through perceived personal relevance, social support, and enjoyment [14,15]. Accordingly, computer game technology has been applied to engage people and to promote health behaviors and clinical outcomes [16,17].

Chronic pain and fatigue complaints constitute a major burden for individuals and societies worldwide [18-20]. Functional somatic syndromes (FSS) are diagnosed by medical specialists when bodily functioning is disturbed, somatic symptoms persist longer than a normal healing process, and conditions cannot be fully attributed to a known conventional disease [21]. A high degree of commonality exists between FSS, wherein central sensitization may be a biological substantiation [22]. FSS can be precipitated by profound life events and cultural factors and maintained by psychosocial factors [20]. Evidence supports the effectiveness of various cognitive and behavioral interventions in primary care settings, or within interdisciplinary rehabilitation programs (IRPs), when “unimodal” psychiatric or physiotherapeutic services do not suffice [20]. Nonetheless, patients were often seen by their general practitioners, but seldom accessed specialized behavioral or multi-modal treatment, and often believed that their complaints are inadequately managed (28%-62%) [17].

### Literature Review

Efficient use of scarce resources and removal of access barriers are important motives for developing CBIs [2]. Results on the effectiveness of computer-based behavioral interventions are promising, but uncertainties regarding their actual usage certainly applies to FSS patients [2,23]. Virtual reality and gaming technologies have been applied for triggering positive emotions, distraction, or graded exposure in rehabilitation and pain management for improvements in physical functioning, pain symptoms, and daily life activities [24,25]. However, there has been no evaluation of the effectiveness of applied gaming as an independently accessible means for delivering behavioral change messages to patients with FSS [16,17,26]. The actual extent and reasons of patient engagement in applied games will largely determine their effect [16]. A better understanding is needed of why CBIs have not been optimally used by which patients with chronic pain and fatigue symptoms to overcome the treatment barriers they face and why integration of applied gaming can offer a partial solution [2,17-19].

### Research Goals

This study aims to explain the usage of applied gaming and provide a comprehensive feasibility description from the perspective of adult patients with chronic pain and fatigue complaints. The opportunity to conduct this study was provided by the planned incorporation of the applied game “LAKA” within a standardized IRP for adult patients with chronic and complex fatigue or pain symptoms in the Netherlands. The primary objective is to explain variation in the demand for applied gaming when offered for voluntary usage during an IRP. Relationships are studied between usage (intentions), behavioral factors, and patient baseline characteristics, including case mix, functional and clinical status, and medical history. In doing so, this study contributes to a better understanding of why applied games are demanded by patients in real health care settings. Second, feasibility was thoroughly described to prepare for a full-scale evaluation in exploring the domains of acceptability, implementation, practicality, and promise for efficacy. Both research goals are reflected in a conceptual framework (see Multimedia Appendix 1) integrating technology acceptance modeling in a feasibility study design [23-27]. Overall, this contribution enables feasible proposals for incorporating and evaluating an applied game for behavioral change within the rehabilitation of patients with complex chronic pain and fatigue complaints.

##  Methods

### Research Design

**Figure 1:**
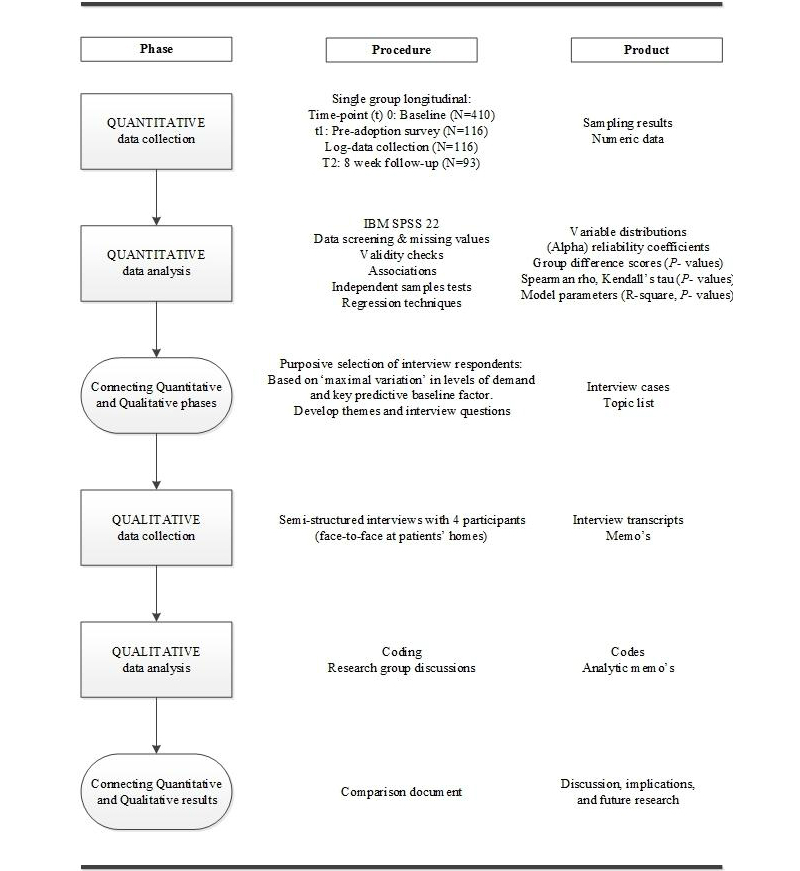
Mixed-methods design overview.

A mixed-methods design was implemented with sequential quantitative (QN) and qualitative (QL) phases [28] (Figure 1). Owing to the availability of adequate quantitative research instruments, an explanatory sequential mixed-methods design worked well for triangulation, illustration, and complementing QN findings with in-depth QL insights and with practically useful information about feasibility [29,30]. The QN phase was prioritized and set up as a longitudinal single-group study of target patient responses to LAKA when offered for voluntary usage during the first 8 weeks of their IRP. The QL phase provided a complementary inductive approach to both research questions. QN and QL phases were mixed in using QN results for the preparation of QL data collection and again when integrating and documenting QN and QL results.

### Recruitment and Data Collection

Ethical approval for the protocol was obtained for this study (at the Psychological Ethical Testing Committee of Tilburg School of Behavioral Sciences, Tilburg University). In total, 410 patients were eligible to start an IRP in 1 of 4 facilities of Ciran, a Dutch rehabilitation center, between 6 January, 2014, and 6 April, 2014 (criteria are listed in Textbox 1) and had given permission to process their diagnostic records for scientific research. Patients in this group were prompted on the day of their first appointment with an email invitation containing information about the nature and consequences of participation in the study, and a link to the “serious gaming page” (see Multimedia Appendix 2). This page guided patients through procedures for software access and additional data collection. Local team leaders were requested to organize face-to-face reminders for patients about the study and the accessibility of LAKA regardless of giving informed consent.

**Inclusion and exclusion criteria for study participants.**

Inclusion criteria:

- Is aged between 18 and 67 years
- Lives in the Netherlands and is proficient in the Dutch language
- Reports the presence of pain for more than 6 months, or fatigue complaints, or a musculoskeletal disease for more than 3 months
- Had received previous primary or secondary health care services without a satisfactory result.
- Reported additional problems on at least 2 of the following problem areas: disturbance of participation, individual or environmental factors, psychological distress, and lack of spiritual well-being

Potential exclusion criteria:

- Presence of medical or psychiatric risk factors (nontreated medical condition, drugs- or alcohol addiction, and suicide risk)
- Presence of third party liabilities

Additional QN data were collected from study participants with two surveys and log-file recordings. The first survey was to be completed within 4 weeks after the invitation and before usage (t1). The second survey (t2) was added to a standard midterm evaluation after 8 IRP weeks, and was to be submitted by the 12th week. Participants’ actual usage behavior had been logged automatically between the pre and post surveys. In the intermediate phase, the focus was on "demand" as the primary subject of the study. Two extreme cases were selected so that a relationship between the most predictive baseline characteristic for the use of LAKA could be clearly illustrated. Two more cases were selected with demand levels that were poorly explained by this “key” predictor and more likely to provide information about omitted factors or counterfactuals. QN results were also used to set up an interview schedule. Purposefully selected participants were invited with a prescripted telephone call after their IRP was finished. Interviews were held during 1- to 1.5-hour home visits and were tape-recorded and transcribed. Field notes were taken, and full transcripts were sent to respondents by email within a week after the interview.

### Intervention

A standardized 16-week IRP was delivered by teams of physiotherapists, psychologists, spiritual counselors, and medical rehabilitation specialists (Textbox 2). A full description of the IRP is provided by Garschagen et al [31].

**Characteristics of the interdisciplinary rehabilitation program (IRP).**

**Tailored:** The program has a modular build-up to match individual care need.

**Outpatient, intensive:** On average, 100 hours delivered by professionals (both individual and group sessions), and 30 self-directed hours.

Integrated program components:

- 45% exercise therapy, graded activity, graded exposure, and education in physiology
- 15% cognitive behavioral therapy
- 15% counseling and guidance in resuming participation in important life domains, such as work, social activities, and family life
- 25% spiritual education

Target outcomes:

- Primary: improvement of well-being [32], and participation in important life-domains (activity and participation domains 4-9 of the International Classification of Functioning) [33]
- Secondary: reduce pain, fatigue, and emotional distress symptoms

As recommended, functional requirements were specified before the modeling and evaluation of LAKA [34]. LAKA delivers skills training with metaphorical simulation elements (encounters) and guided exercises for focused attention and open awareness [35]. These elements are interspersed with images of real-world environments, immersive mini-games, and in-game debriefings for “transferring” new insights beyond the virtual world (Textbox 3). Basic information on functional specifications and playability feedback are described and illustrated with screenshots and trailers in Multimedia Appendix 3. A Windows version of LAKA was offered for usage wherever and whenever convenient, without support from health professionals. No recommendation for a minimum or maximum amount of usage was given, and no prompts or reminders were sent. On the one hand, it was expected that many target patients would not use LAKA because of this noncommittal mode of delivery. On the other hand, this variation was desired to discover explanations and practical suggestions that generally apply for delivery in open and clinical settings.

**LAKA design.**

- **Problem addressed:** In complex cases, suffering associated with FSS has an intrusive impact on patients’ existence and their interactions with caregivers, family, or friends [36].
- **Design team:** The design team involved entrepreneurs, researchers, a scriptwriter, game designers, artists, programmers, audio experts, voice-actors, and IT specialists.
- **Stakeholder involvement:** Feedback sessions (on functional specifications, theoretical model, and prototypes) involved experts (in behavioral science, medical technology, and spirituality), and critical users.
- **Genre:** Single-player adventure game.
- **Goal (of the game):** LAKA was designed to provide skills training in “spiritual” practices. Practices are focused on behavioral qualities that are associated negatively with negative emotions and positively with psychological well-being: “generosity,” “moral discipline,” “patience/forbearance,” “enthusiastic energy,” and “mental stability.” Accordingly, the design includes the delivery of various behavioral change techniques integrated in an immersive simulation environment.
- **Main challenges (in the game):** Identify with a personal Avatar and engage in a quest. The story is about an Avatar, who learns about “the art of living” while traveling the world after a significant deterioration of his/her condition. Tasks primarily entail the consideration and evaluation of response options in virtual “encounters” with nonplaying characters.
- **Application components:** Introduction, 4 training modules (or travel “destinations”: London, Turkey, Asia, Africa) with recurrent components, and a celebratory end.
- **Duration:** Completing the game from start to end takes about 2.5 hours.
- **Game controls:** Interaction design and controls (with computer mouse and keyboard) were designed for ease of use. Progression in the game does not depend on gamer performance or skill.
- **Graphics:** Mixed 2D and 3D graphics with comical cut scenes.
- **Sound:** Voice-overs and music convey emotions and atmosphere.
- **Platform:** Personal computer or laptop (MS Windows version).
- **System requirements:** Windows XP or beyond, a 6 gigabytes hard drive, 1 gigabyte memory, and a stable Internet connection.
- **Accessibility:** Via the “serious gaming” Web page by downloading, or by following instructions for picking up a digital versatile disk at local facilities.

### Measures

#### Demand, Demand Factors, and Other Feasibility Domain Outcomes

Objective indicators of demand were based on automatic data logs of participant activities: “frequency” (number of days on which progress was logged), “duration” (sums of time intervals between logins and subsequent data logs), and “progress” (the number of completed encounters). Demand was rated subjectively, before and after usage, by the extent of agreement (1, completely disagree, to 7, completely agree) with 3 statements about their current behavioral intention (BI) to use LAKA during their IRP [24,25]. Seven-point Likert scales were also used to assess behavioral factors, including performance and effort expectancies, social influence, perceived behavioral control, computer anxiety and self-efficacy, hedonic motivation or enjoyment, habit, and trust [23,24,37]. Multimedia Appendix 4 contains details about all survey measures, including variable definitions, items, validity, and reasons why behavioral factors may be relevant [38-46]. Practicality was indicated by counting logins of participants with positive behavioral intentions (BIs at t1 ≥5) as evidence of success in obtaining the software, installation, and running the application. Study and usage attrition were interpreted as indicators of the degree of implementation. Acceptability was operationalized as postusage perceived appropriateness in enjoyment, ease, and knowledge improvement in participants who completed at least the first module of the game.

#### Baseline Measures

Retrieved baseline variables were categorized into case mix, functional status, clinical status, and previous treatment variables (see Multimedia Appendix 4). Case-mix variables included sex, age, education level, environmental issues, and treatment facility. Preferred coping styles were measured with the Utrecht Coping List (UCL). Functional status variables included the duration and course of health complaints, employment status, absenteeism, and 1-item general subjective health. Pain intensity was assessed with an 11-point Numerical Rating Scale (NRS) [47]. The Checklist Individual Strength was used to assess fatigue dimensions [48]. Clinical status variables included a categorization of the chronic symptom patterns by a rehabilitation specialist (primarily a fatigue or musculoskeletal or other pain condition). Body mass index (BMI) and blood pressure were measured during physical examination. Psychopathology dimensions were assessed with the Dutch 90-item Symptoms Check List (SCL-90) [49]. The Pain Coping and Cognitions List and Tampa Scale of Kinesiophobia were used to measure pain coping and cognitions [50,51]. Finally, patients indicated previous specialized treatments and current medication intake.

###  Data Analyses

#### Data Exclusion

Cases were list-wise deleted before analysis if the proportion of missing observations was <5%, or handled by predicting 5 data imputations for each empty cell through regression of all variables in the dataset (using the MCMC algorithm). All full-case QN findings presented as marked results are supported by pooled results.

#### Participant Statistics

Characteristics of eligible patients, study participants, and participants who logged into the game (players) are described by descriptive statistics and frequencies. Chi square and Mann-Whitney *U* tests were used to compare baseline level characteristics between study participants and participants who logged into LAKA, versus eligible patients that were not included in those groups. Similarly, differences were tested between participants who logged in versus participants who did not log in.

#### Process Analyses of Demand and Feasibility Descriptions

All feasibility outcomes of applied gaming during the first 8 weeks of the IRP are indicated with descriptive statistics and line graphs. Association measures (Spearman *ρ* and Kendall τ statistics) between baseline characteristics, behavioral factors, and feasibility outcomes were calculated and tested for significance. Moreover, multiple ordinary least squares regression analyses were performed for the sequential identification of important constituent factors of behavioral intention at t1, to explore whether effects of behavioral factors differed between subgroups of patients (see Multimedia Appendix 5), and to test if marked associations between baseline characteristics and behavioral intentions were mediated by behavioral factors [52].

#### Qualitative Data Analysis

Interview transcripts were coded by one author (MV) using a software package: MAXQDA 11 (VERBI GmbH) [53]. In the first coding step, all text fragments about the specified interview topics were labeled with short statements that corresponded with contextual meanings. A second author (MJ) independently repeated this first coding step for one interview. These “first order” codes were compared and discussed between MJ and MV to align and refine the coding procedure. In a second coding step, more abstract categories were generated. Throughout this process, first-order codes and emergent categories were constantly compared and hierarchically structured as a means for critical appraisal and to avoid imposing preconceived ideas on the QL data. Finally, categories were related to one another by designating them as context factors, conditions (barriers or facilitators), events or interactions, or consequences.

#### Mixing Quantitative and Qualitative Results

In connecting QL and QN findings, codes and statistics were provided for comparison for both research questions. QN results were deemed notable for comparison with QL findings if *P* values were below .05. Subsequently, 3 researchers (MV, MJ, and HV) discussed and determined points of convergence, divergence, or complementariness between QN and QL findings. In doing so, observations were summarized to determine which, and to what extent, remarkable and solid QN findings were clearly illustrated and triangulated. Moreover, the point at which qualitative data collection was stopped was determined on the basis of saturation with respect to illustrations of behavioral factors and the role of a key predictive baseline characteristic for usage in early stages.

##  Results

### Participant Statistics

**Figure 2:**
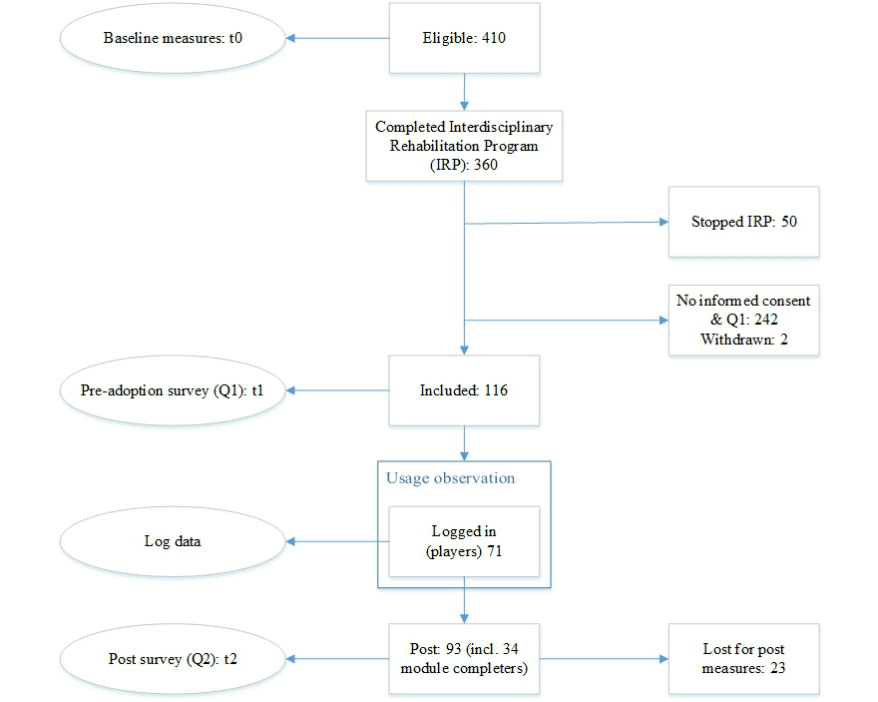
Flow diagram of study participants.

Of the 410 invited eligible patients, 32.2% provided informed consent and completed the first additional survey (Figure 2). The 84 patients who reported why they did not wish to participate mentioned “other obligations” (23), “facilitative problems” (14), “no intention to use the intervention” (14), “not enough energy or concentration” (13), “no interest to participate in the research” (10), “bodily complaints” (8), or “other reasons” (2). One patient withdrew because of a broken computer, and one for experiencing excessive hindrance in attempting to use an unsupported Web browser. The second questionnaire was submitted by 93 participants (80.2%).

Study participants’ average age was 44.4 years (SD 10.8 years; range 21-63 years); 71% were female (Table 1). Sixty-nine participants were completely absent from work. The average duration of absenteeism was 157 days (SD 223.0), with a median slightly more than 100 days. Forty-seven participants (40.5%) reported moderate to severe pain (5-10), and 108 experienced problematic fatigue. Average scores for depressive (42.9, SD 11.4) and anxious (22.2, SD 8.2) symptoms were high. Participants had been regularly surfing the Internet, but only 46 patients (39.7%) had been playing on a computer over the past year. No statistically significant differences between participants and nonparticipants were found for case-mix variables. However, patients with more severe pain symptoms were underrepresented in the sample (Table 2). The group of 71 patients who actually logged in (players) reported relatively higher scores for coping through active engagement and social support seeking, lower scores for pain coping, and fewer environmental issues. The proportion of patients who had received specialist treatment for their current complaint was lower among players than among nonplayers (χ^2^_1_=4.1; *P*=.042; not in Table 2).

Four interview respondents were selected based on their combination of scores for coping by active engagement and demand (Table 3). Open questions were asked to introduce and focus on topics (see Multimedia Appendix 6). Two topics addressed the primary research question, namely, “initial response” to the digital game offering (topic 1) and patient “experiences” throughout their interactions with LAKA (topic 2). Topic 2 and “suggestions for improvement” (topic 3), served to collect complementary information on feasibility domains. After a first round of mixing, 4 interviews was deemed sufficient to provide clear illustrations of the most notable QN explanations for demand.

**Table 1:** Characteristics of study participants (N=116).

Characteristic^a^		N (%)
**Demographics**		
Sex		
	Female	71 (61.2)
Age, years		
	<35	23 (19.9)
	35-45	30 (25.9)
	45-55	41 (35.3)
	55-67	22 (19.0)
Education level ISCED^b^		
	Primary or less	32 (25)
	Lower to postsecondary	44 (37.9)
	Tertiary and posttertiary	40 (34.5)
	Missing	3 (2.6)
		
**Functional status**		
Employment in paid work		
	Full-time	49 (42.2)
	Part-time	52 (44.8)
	None	15 (12.9)
Absenteeism		
	Not	15 (26.1)
	Partially	17 (14.8)
	Completely	69 (59.1)
Duration of absenteeism for present somatic symptoms		
	<3 months	31 (26.7)
	0-3 month	41 (35.3)
	3-6 months	22 (19.0)
	6-12 months	14 (12.1)
	1-2 years	6 (5.2)
	>2 years	2 (1.7)
Symptom duration		
	<3 months	3 (2.6)
	3-6 months	11 (9.5)
	6-12 months	30 (25.9)
	1-2 years	27 (23.3)
	>2 years	45 (38.8)
Pain NRS^b^		
	No pain (0)	18 (15.5)
	Mild pain (1-4)	51 (44.0)
	Moderate pain (5-7)	36 (31.0)
	Severe pain (7-10)	11 (9.5)
Fatigue		
	No fatigue (NRS^b^=0)	2 (1.7)
	Not problematic (CIS^b^ ≤76)	6 (5.3)
	Problematic (CIS >76)	108 (94.7)
CIS subjective fatigue^c^		
	Above average	50 (43.1)
CIS physical activity^c^		
	Below average	67 (58.8)
		
**Clinical status**		
Primary diagnosis		
	Chronic musculoskeletal disorder	21 (18.1)
	Chronic pain	17 (14.7)
	Chronic fatigue	78 (67.2)
SCL-90^b^ depression^d^		
	Below average (16-31)	24 (20.7)
	Above average (32-35)	7 (6.0)
	High (36-52)	63 (54.3)
	Very high (≥53)	22 (19.0)
SCL-90 anxiety^d^		
	Below average (10-17)	42 (36.2)
	Above average (18)	7 (6.0)
	High (19-28)	39 (33.6)
	Very high (≥29)	28 (24.1)
		
**Previous treatment**		
Medical specialist treatment		
	Yes	70 (60.3)
Medication usage		
	Yes	80 (69.0)
	Missing	1 (.9)
		
**Previous use of similar technology**		
Habit of frequent Internet usage with a PC or laptop		
	On 6-7 days per week	84 (72.4)
	On 3-5 days per week	22 (19.0)
	On 1-2 days per week	9 (7.8)
	On <1 day per week	1 (0.9)
Experience of digital game play		
	Never played a digital game	37 (31.0)
	More than a year ago	33 (28.4)
	Less than a year ago	14 (12.1)
	Less than a month ago	32 (27.6)
Habit of frequent digital game play		
	One or more times per month (and less than a month ago)	29 (25)

^a^A selection of individual baseline characteristics is presented to facilitate comparison with previous evaluations of behavioral interventions for FSS patients [54,55].

^b^ CIS: Checklist Individual Strength, ISCED: International Standard Classification of Education (according to which highest education levels [Dutch system] were rescaled [low = 0-1, middle = 2-4, high = 5-6]) [56], NRS: Numerical Rating Scale, SCL: Symptom Checklist.

^c^For all participants (2 missing values were ignored; N=114). In comparison with the average in a population of patients with chronic fatigue syndrome [57].

^d^Compared with a population of Dutch patients with chronic pain [49].

**Table 2:** Overview of independent samples tests.

Variable	Participants (N=116) vs nonparticipants^a^	Players (N=71) vs nonplayers or nonparticipants^a^
	Mean (SD)/frequency (%), *P* value of test statistic^b^	Mean (SD)/frequency (%), *P* value of test statistic^b^
**Case mix**		
Female (dit.)^b^	71 (61.2), .48	45 (63.4), .92
Age^b^	44.4 (10.8), .91	44.1 (11.3), .88
Education level	3.3 (1.3), .12	3.3 (1.3), .40
UCL^c^ active engagement	17.6 (4.0), .53	18.5 (3.9), .02
UCL passive responding	14.3(3.7), .29	14.0 (4.0), .11
UCL social support seeking	13.9 (4.0), .06	14.1 (4.0), .03
UCL comforting thought	12.0 (2.7), .74	12.4 (2.7), .38
Environmental issue (dit.)	61 (53.4), .07	34 (47.9), .02
Location A (dit.)	39 (33.6), .39	18 (25.4), .30
Location B (dit.)	27 (23.3), .41	19 (26.8), .89
Location C (dit.)	29 (25.0), .53	18 (25.4), .59
Location D (dit.)	21 (18.1), .45	16 (22.5), .64
**Clinical status and functioning**		
Body mass index	27.1 (5.8), .32	27.4 (5,5), .17
Indication for chronic fatigue (dit.)	78 (67.2), .045	54 (76.1), .002
Indication for musculoskeletal disorder (dit.)	21 (18.1), .16	9 (12.7), .03
Indication for chronic pain (dit.)	17 (14.7), .37	8 (11.3), .14
Symptom duration	Median >2 years, .75	Median >2 years, .34
Symptom recurrence (dit.)	74 (63.8) .75	44 (62.0), .68
Symptom deterioration (dit.)	69 (59.5), .04	43 (60.6), .20
Paid work (dit.)	101 (87.1), .12	62 (87.3), .23
SCL^-^90^c^total	206.9 (50.7), .70	206.3 (51.2), .94
SCL-90 sleeping problems	9.1 (3.3), .18	9.0 (3.4), .23
SCL-90 hostility	11.3 (4.5), .38	11.1 (4.1), .69
SCL-90 interpersonal sensitivity	34.9 (12.7), .58	33.9 (12.4), .63
SCL-90 insufficiency	26.0 (7.0), .73	26.6 (6.9), .27
SCL-90 somatization	30.7 (8.3), .86	31.3 (7.7), .40
SCL-90 depression	42.9(11.4), .69	41.8 (11.7), .55
SCL-90 anxiety	22.2 (8.2), .86	22.4 (8.5), .99
SCL-90 agoraphobia	11.0 (5.4), .60	11.5 (6.0), .73
		
	N=47^d^	N=27^d^
Pain NRS^c^	6.5 (1.3), .046	6.6 (1.4), .21
PCCL^c^ internalization	3.2 (.7), .09	3.3 (.7), .18
PCCL pain coping	2.8 (.8), .06	2.7 (.8), .009
PCCL catastrophizing	3.6 (.8), .04	3.6 (.8), .11
TSK^c^	36.7 (6.8), .80	36.4 (7.1), .64
	N=108^e^	N=69^e^
CIS^c^ subjective fatigue	50.5 (6.0), .32	50.4 (5.6), .85
CIS concentration	26.3 (8.1), .59	26.0 (8.4), .57
CIS motivation	21.3 (6.1), .59	21.4 (6.1), .63
CIS physical (in)activity	17.2 (4.0), .83	16.9 (4.1), .30
CIS total score	110.1 (14.8), .99	109.4 (14.1), .64
	N=101^f^	N=62^f^
Part-time work (dit.)	52 (53.1), .72	33 (46.5), .57
Weekly work hours	31.6 (11.9), .21	29.8 (15.4), .12
Absent (dit.)	86 (74.1), .94	54 (87.1), .60
UBOS-a^c^ burnout (dit.)	26 (22.4), .33	16 (22.5), .48
UBOS mental exhaustion	3.9 (1.5), .72	4.0 (1.5), .47
UBOS distancing	2.3 (1.5), .81	2.4 (1.6), .99
UBOS work competence	4.0 (1.2), .20	4.1 (1.1), .08
	N=86^g^	N=54^g^
Partially absent (dit.)	17 (14.7), .31	13 (24.1), .94
Sick leave duration	159.8 (223.4), .91	150.4 (15.4), .36
**Previous treatment**		
Medication intake (dit.)	70 (60.3), .48	39 (58.2), .06
Previous specialist treatment (dit.)	81 (69.8), .50	47 (66.2), .83

^a^These comparisons were chosen to inform about study sample profiles and how successful the implementation was in recruiting representative subsamples for exploring “within-group” variation in demand. The players versus nonplayers comparison did not yield more remarkable differences.

^b^N (%) and *P* value of chi-square if variable is dichotomous (dit.); median (N) or mean (SD) and *P* value of Mann-Whitney *U* test if variable is an ordinal or a ratio scale value.

^c^CIS: Checklist Individual Strength, PCCL: Pain Coping and Cognitions, SCL: Symptom Checklist, TSK: Tampa Scale of Kinesiophobia, UBOS-a: Utrecht Burnout Scale labor (a) version, UCL: Utrecht Coping List.

^d^Subsample of participants with moderate or severe pain, ^e^with problematic fatigue, and ^f^with paid work and of those ^g^absent from work.

**Table 3:** Characteristics of interview respondents.

Characteristic	Respondent #1^a^	Respondent #2	Respondent #3	Respondent #4
Usage (session days, encounters)	3, 20	0, 0	1, 1	2, 4
Behavioral intention	6	1	4	7
UCL active engagement^a^	Very high	Very low	Very high	Average
Sex	Male	Male	Female	Female
Age, years	35	57	62	54
Work status	No paid work	Fully absent for 97 days	Fully absent for 287 days	Present at work
CIS fatigue severity^c^	Problematic	Above-average CFS^b^	Above-average CFS	Problematic
Pain NRS	3	1	3	0
SCL^d^ anxiety	Average	<Average	High	High

^a^Cases were identified by inspection of a bivariate scatterplot displaying the most predictive individual baseline characteristic on the x-axis; frequency of usage on the y-axis; and marking dots representing negative (<3), neutral (3-5), and positive (>5) behavioral intentions at t1.

^b^Levels of active engagement within the sample are similar to healthy worker population levels. Norm scores are slightly different for males and females.

^c^As compared to average fatigue severity in a sample of patients diagnosed with chronic fatigue syndrome.

^d^CIS: Checklist Individual Strength, NRS: Numerical Rating Scale, SCL: Symptom Checklist, UCL: Utrecht Coping List.

### Process of Demand

#### Direct effects

Actual usage indicators measured at preadoption were associated with behavioral intention at a moderate level at t1 (*ρ*=.527-.546), and weakly at t2 (*ρ*=.260-.273). Behavioral intention was associated with various behavioral factors measured at t1 (Table 4). Effects of perceptions on performance, social norms, and knowledge improvement became stronger over time and with exposure to LAKA. Expectancies of effort and performance independently shared a significant amount of variance with behavioral intention at t1 (Table 5). Second step models were superior to the core model if supplemented with hedonic motivation or habit, but not if other factors were added. Inclusion of hedonic motivation weakened the effects of core factors such that performance expectancy was no longer a significant factor for behavioral intention.

If asked openly for an explanation of their own initial response to the offer to play LAKA during their IRP, respondents first explained their affect or attitude at that time, such as their openness to try the intervention. These feelings were substantiated by memories of previous experiences with computer game play. Those who felt positive about using the game did not experience barriers in concerns about privacy, usefulness, opinions of others, or a lack of resources. Nonetheless, respondents desired an explanation about how the intervention could bring about personal benefit.

I do like games. I have them myself… I'm just going to see what it is. Not immediately: no, I do not join in… I was just open minded… With two or three people I have talked about it (eds.: study, LAKA), and they said: the only thing for which I look at the computer, is to see what time I should be here. For other things; let me know…

Respondent #4

I did not recognize a goal… If there was a little more explanation, then I would have probably played ... and especially if it helps.

Respondent #2

Various associations between case-mix variables and demand indicators were marked (Table 6). Frequency of coping by active engagement and comforting thought were positively related to demand before exposure. Associations between demand indicators and social support seeking increased by exposure. BI was significantly lower for participants treated in location A, whereas participants treated in location D progressed more within the game. BI measured after 8 weeks was associated negatively with education level and positively with age. Notable differences in demand by functional and clinical status variables were also observed. LAKA was played more frequently by participants who reported partial absenteeism from work and lower pain coping scores. Higher usage was registered for players with higher levels of perceived competence in their job, symptom deterioration, higher pain intensity, lower internalization, and fewer symptoms of anxiety at baseline.

**Table 4:** Associations between demand indicators and behavioral factors.

Behavioral factor	BF t1 with BI t1^a^ N=115^b^ (*ρ* or τ, *P* ^*c*^)	BF t1 with BI t2 N=92 (*ρ* or τ, *P*)	BF t2 with BI t2 N=32 (*ρ*, *P*)
Performance expectancy	.33, <.001	.19, .08	.59, <.001
Expected ease	.42, <.001	.10, .37	.35, .045
Social influence	0.14, .13	.17, .11	.42, .01
Perceived behavioral control	.33, <.001	.04, .71	.22, .22
Trust	.31, .001	.21, .049	.53, .001
Hedonic motivation	.54, <.001	.43, <.001	.61, <.001
Computer anxiety	−.27, .003	.10, .35	
Computer self-efficacy	.22, .02	.32, .002	
Habit (dichotomous)	.22^c^, .007	.06^c^, .53	
Perceived knowledge improvement			.77, <.001

^a^BI: behavioral intention, BF: behavioral factor, t: time-point.

^b^Pairwise deletion: one respondent submitted an unfinished web-survey at t1.

^c^Kendall τ (for dichotomous variable) or Spearman *ρ* (for other variables), *P* value.

**Table 5:** Parameters and models fit of multiple regression for constituent factors of behavioral intention at preadoption.

Parameters (N=115^a^)	Model 1: Core TAM^b^ beta (*P* value)	Model 2: UTAUT^b^ beta (*P* value)	Model 3: Core+HM^b^ beta (*P* value)	Model 4: Core+HB^b^ beta (*P* value)
Constant	.84 (.15)	.34 (.63)	.89 (.10)	.69 (.23)
PE^c^	.40 (.002)	.35 (.009)	.04 (.79)	.46 (<.001)
EE^c^	.52 (<.001)	.51 (<.001)	.27 (.02)	.47 (.001)
SI^c^		.15 (.09)		
PBC^c^		.06 (.63)		
HM^c^			.59 (<.001)	
HB^c^				.63 (.02)
				
	R^2^ (*P* of ΔR^2^)^c^	R^2^ (*P* of ΔR^2^)^d^	R^2^ (*P* of ΔR^2^)^d^	R^2^ (*P* of ΔR^2^)^d^
Model	.34 (<.001)	.36 (.22)	.43 (<.001)	.38 (.02)

^a^Observations of 1 incomplete case were listwise deleted.

^b^EE: effort expectancy, HB: habit, HM: hedonic motivation, TAM: technology acceptance model, UTAUT: unified theory of acceptance and use of technology, PBC: perceived behavioral control, PE: performance expectancy, SI: social influence.

^c,d^ *P* of ΔR^2^ is the *P* value of variance explained by the model over ^c^a constant-only model, or ^d^over model 1.

**Table 6:** Associations between baseline characteristics and demand indicators.

Baseline variable	BI t1 *ρ* or τ, *P* ^a^	Session days *ρ* or τ, *P*	Session days *ρ* or τ, *P*	Time spent *ρ* or τ, *P*	Progress *ρ* or τ, *P*	BI t2 *ρ* or τ, *P*
**Case-mix**						
	N=116	N=116	N=71	N=71	N=71	N=93
Female (dit)^a^	−.04, .59	−.03, .77	−.10, .34	−.10, .29	−.13, .22	−.08, .38
Age	−.10, .28	−.02, .84	−.05, .69	.03, .78	−.00, .97	.25, .02
Education level (ISCED)^b^	−.05, .60	−.12, .18	−.21, .08	−.24, .048	−.24, .048	.28, .006
UCL^b^ active engagement	.25, .008	.30, .001	.23, .06	.13, .29	.13, .28	.11, .31
UCL passive responding	−.09, .32	−.16, .08	−.19, .12	.02, .86	−.02, .89	.02, .82
UCL social support seeking	.09, .37	.19, .045	.23, .052	.20, .08	.24, .04	.08, .43
UCL comforting thought	.20, .03	.19, .04	.11, .36	.09, .44	.09, .48	.05, .62
Environmental issue (dit.)	−.15, .06	−.03, .13	.13, .22	.07, .48	.10, .34	.06, .50
Location A (dit.)	−.17, .03	−.22, .01	−.13, .22	−.18, .06	−.19, .07	−.02, .86
Location B (dit.)	.07, .42	.01, .93	−.15, .15	−.14, .17	−.18, .08	−.091, .31
Location C (dit.)	.07, .37	.06, .47	.15, .18	.12, .23	.14, .18	.030, .74
Location D (dit.)	.06, .46	.19, .03	.15, .17	.21, .03	.24, .02	.084, .35
						
**Clinical and functional status**						
	N=116	N=116	N=71	N=71	N=71	N=93
Body mass index	.05, .64	.13, .17	.06, .60	.06, .65	.11, .39	.10, .34
Indication chronic fatigue (dit.)	.06, .48	.07, .43	−.18, .09	−.18, .06	−.16, .12	.15, .10
Indication musculoskeletal (dit.)	−.02, .78	−.05, .56	.16, .15	.10, .30	.10, .31	−.16, .07
Indication chronic pain (dit.)	−.05, .53	−.04, .69	.08, .44	.14, .16	.10, .31	−.02, .83
Pain intensity NRS^b^	−.03, .79	.15, .12	.29, .02	.28, .02	.29, .02	.05, .63
Symptom duration	−.05, .58	−.08, .40	−.01, .92	.01, .94	.03, .79	−.04, .70
Symptom recurrence (dit.)	−.02, .74	−.05, .53	−.08, .49	−.12, .23	−.09, .37	.03, .78
Symptom deterioration (dit.)	.05, .51	.14, .09	.21, .054	.25, .01	.20, .048	.13, .17
Paid work (dit.)	.04, .64	.03, .77	.07, .54	.03, .75	−.00, .98	−.10, .30
Part-time (dit.)	.07, .38	.05, .54	.09, .43	.04, .67	.03, .78	.08, .37
Weekly work hours	.09, .35	.05, .60	.02, .87	.04, .75	.03, .84	−.16, .13
Work absence (dit.)	−.01, .89	.02, 87	−.07, .52	.02, .88	−.01, .97	.05, .64
SCL^b^ total	−.08, .41	−.11, .23	−.20, .09	−.06, .63	−.12, .30	−.05, .63
SCL sleeping problems	−.12, .19	−.10, .29	−.09, .48	−.05, .71	−.10, .40	−.01, .96
SCL hostility	−.09, .32	−.01, .94	−.03, .83	.01, .97	−.03, .80	.07, .47
SCL interpersonal sensitivity	−.14, .14	−.13, .17	−.16, .19	−.04, .73	−.06, .61	−.01, .96
SCL insufficiency	.03, .74	.03, .78	−.13, .29	−.02, .86	−.09, .44	−.01, .90
SCL somatization	.01, .94	.09, .36	.02, .89	.07, .56	.01, .92	.02, .88
SCL depression	−.11, .25	−.17, .07	−.18, .13	−.03, .78	−.09, .44	−.11, .28
SCL anxiety	.00, .98	−.13, .18	−.28, .02	−.15, .23	−.21, .08	.04, .74
SCL agoraphobia	.02, .83	−.06, .50	−.29, .02	−.14, .25	−.19, .11	−.07, .53
						
	N=47	N=47	N=27	N=27	N=27	N=38
PCCL internalization	.14, .37	−.11, .46	−.36, .07	−.42, .03	−.48, .01	−.06, .73
PCCL pain coping	−.03, .87	−.35, .02	−.28, .16	−.25, .21	−.26, .19	−.01, .95
PCCL catastrophizing	.03, .83	.02, .92	.16, .42	.30, .14	.25, .21	.03, .84
TSK kinesiophobia	−.08, .61	−.08, .58	.09, .67	.26, .20	.23, .25	−.08, .64
						
	N=108	N=108	N=69	N=69	N=69	N=86
CIS^b^ subjective fatigue	−.04, .66	−.09, .38	−.07, .55	−.02, .90	−.10, .42	.03, .80
CIS concentration	.07, .49	−.10, .30	−.15, .22	−.05, .67	−.09, .47	.15, .16
CIS motivation	−.08, .44	−.05, .59	−.13, .29	−.04, .72	−.10, .41	.19, .08
CIS physical or inactivity	.02, .81	−.05, .60	.02, .90	.13, .27	.09, .48	−.04, .75
CIS total score	−.02, .82	−.12, .22	−.18, .14	−.06, .64	−.13, .29	.16, .15
						
	N=101	N=101	N=62	N=62	N=62	N=93
UBOS-a burnout (dit.)	−.01, .95	−.10, .30	−.23, .050	−.17, .10	−.20, .06	.04, 67
UBOS-a mental exhaustion	.03, .77	.01, .90	−.08, .55	−.11, .42	−.11, .38	.11, .35
UBOS-a distancing	.10, .93	−.08, .40	−.19, .15	−.16, .22	−.20, .12	.10, .36
UBOS-a work competence	.15, .14	.19, .052	.29, .02	.27, .03	.32, .01	−.14, .22
						
	N=86	N=86	N=54	N=54	N=54	N=69
Partially vs. fully absent (dit.)	.06, .55	.26, .01	.27, .03	.18, .11	.20, .09	.09, .40
Sick leave duration	.21, .054	.13, .24	.02, .91	.01, .96	.00, .98	−.10, .41
						
**Previous treatment**						
	N=116	N=116	N=71	N=71	N=71	N=93
Medication intake (dit.)	−.09, .44	−.15, .09	−.13, .22	−.10, .31	−.11, .27	.02, .79
Specialist treatment (dit.)	−.03, .72	−.06, .53	.16, .16	.08, .41	.15, .15	.09, .35

^a^ *ρ*: Spearman *ρ* statistic was calculated when both variables had interval or ratio measurement levels, τ: Kendall τ statistic was calculated for dichotomous level independent variables (dit.) *P*: *P* value of test statistic.

^b^CIS: Checklist Individual Strength, ISCED: International Standard Classification of Education Level, NRS: Numerical Rating Scale, PCCL: Pain Coping and Cognitions, SCL: Symptom Checklist, TSK: Tampa Scale of Kinesiophobia, UBOS-a: Utrecht Burnout Scale labor (a) version, UCL: Utrecht Coping List.

Three interview respondents who exposed themselves to LAKA explained their level of engagement by witnessing that game tasks were welcome challenges in early stages of a rehabilitation process. However, patient users’ attention shifted away from gaming tasks toward the pace (slow) and structure of the game when their confidence and engagement in “real-life” roles increased (eg, noticing that selecting preprogrammed alternatives is not as complex as responding in real life, and purposively selecting “bad” responses to explore the “rules” that guide scenarios). Disengagement was also explained by the belief of being incapable to perform a certain task.

At the time of the program… I was on sick leave. What could I do? I really had time for the computer, and no energy for anything else... When I stopped, it was enough for me. The game is too slow for me… For my energy that I've built up again... I started working again. I'm going to a sports club. Yes, my life, my rhythm, is different... I have no time.

Respondent #3

In work, I am constantly adjusting to people. So for me it did not really matter… I have an ADHD problem. So, attention exercises are a disaster for me. I have no patience for that… The first time I went on to see where I got stuck when I was just giving ‘wrong’ answers… Occasionally, when you had to wait, I was like: come on, hurry.

Respondent #1

#### Moderation and Mediation Effects

Performance expectancy was a significantly stronger predictor of behavioral intention at t1 in patients primarily diagnosed with chronic fatigue rather than a chronic pain condition (beta=.98; *P*<.001), and high levels of depressive symptoms (beta=.91; *P*=.006). The relationship between social influence and behavioral intention was affected negatively by the more than 6-month absenteeism (beta= .61; *P*=.01). Daily Internet usage over the past year strengthened the positive effect of hedonic motivation on behavioral intention at preadoption (beta=.63; *P*=.001). The negative association between computer anxiety and behavioral intention was significantly weaker in participants younger than 45 years (beta=.42; *P*=.009). Mediation analyses showed that perceived behavioral control mediated the effect of active engagement on behavioral intention at preadoption, but did not mediate the effect of active engagement on the presence of a log-in.

Focusing on individual differences in coping with the delivery of LAKA during interviews yielded self-descriptions by patients, which varied between being “curious, a gamer, and capable” to play versus being neither a “games person” nor an “early adopter” and believing that computer games are difficult to play.

Anyway, I am someone who games a lot ... Did not doubt about being able to play it. I am someone who wants to follow and keep up with things ... There are buttons, and all the buttons I want to have tried them at least once.

Respondent #1

Most games that happen to PCs, such as Tetris and things like that… That is under time pressure … No, that does not attract me and I cannot do that ... I'm not the pioneer to go on my own.

Respondent #2

### Feasibility Description

#### Demand

**Figure 3:**
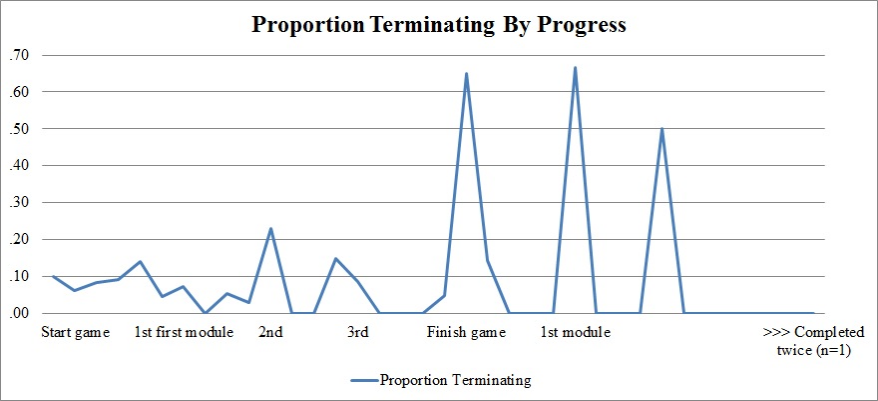
The proportions of players who stopped using LAKA at certain stages of progress.

At the preadoption stage, most participants had a moderately positive intention to use LAKA over the next 4 months in addition to their scheduled IRP activities (Table 7). Nine participants (7.8%) with low initial behavioral intention (2 or lower) were statistical outliers, but were not excluded from further analyses. BI decreased over the course of 8 weeks. On average, players completed 8 encounters, which equals 2 of 4 modules in total. The first module of the game was completed by 40 patients (56.3%). Twenty players (28.2%) completed the game from start to end. A line graph (Figure 3) shows that players were more likely to stop using the game when they headed for a new game, module, or element.

#### Implementation and Practicality

**Figure 4:**
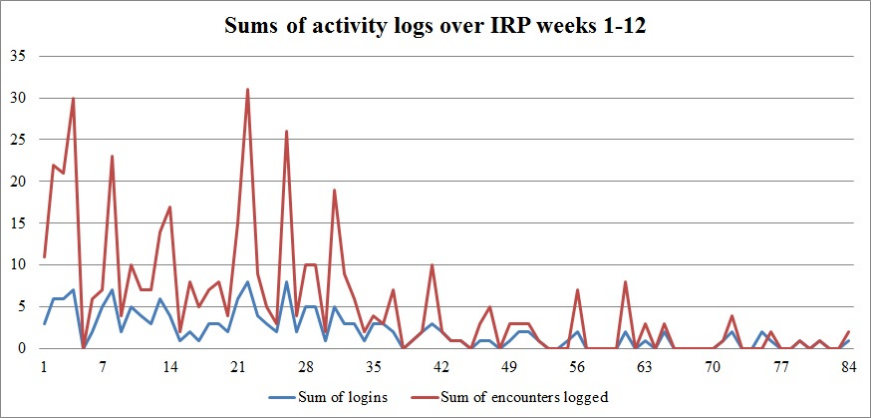
Sums of logged in game activities throughout the first 12 weeks of patients' IRPs.

**Figure 5:**
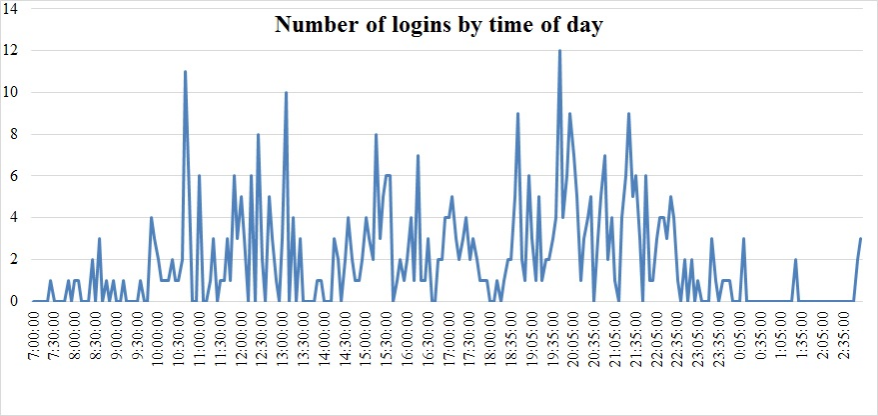
Number of logins by participants by time of the day.

Of 85 participants, 59 (69.4%) with a positive intention to use (BI ≥5) logged in successfully. At treatment facility A, 12 of the 24 willing participants logged in (50%), which is significantly less (χ^2^_1_=5.9; *P*=.015) than the proportions of participants at the other 3 locations (70.6%-81.0%). Players who possessed more computer platforms (ie, a tablet, a console, a mobile phone) progressed less within the game (*ρ*= .39; *P*=.001). Most activity was recorded during the first 4 weeks of participants’ IRPs (Figure 4). When playing at home, participants logged in at different times during the day, but mostly after 6 pm (Figure 5).

**Table 7:** Descriptive results of demand level assessment.

Demand indicator	N	Mean	Median	SD^a^	Min	Max
BI^a^ at t1	116	5.1	5	1.4	1	7
BI at t2	93	3.5	4	2.0	1	7
Session days	71	1.8	2	1.4	0	6
Time spend	71	1:14:40	0:52:25	1:07:42	0:00:00	4:22:27
Progression	71	8.1	7	7.3	0	32

^a^BI: behavioral intention, SD: standard deviation.

#### Acceptability and Potential for Efficacy

Postusage perceived ease, rated by those who played the first module of LAKA, was moderately high (mean 5.4/7 (SD 1.54); median 6/7) and varied positively with baseline active engagement (*ρ*=.45; *P*=.007), motivation (*ρ*=.57; *P*=.001), and indication of a chronic musculoskeletal disorder (*P*=.008). Perceived enjoyment levels were moderately positive (mean 4.6 (SD 1.7)), and positively associated with baseline concentration problems (*ρ*=.44; *P*=.01). Postusage perceived knowledge improvement (mean 4.6/7 (SD 1.8)) was lower in participants with a higher BMI (*ρ*= .42; *P*=.02).

Interview respondents who played LAKA (respondents 1, 3, and 4) believed that it was a suitable program component. A variety of game elements were appointed that were liked. Furthermore, tasks were quickly understood, taken seriously, and experienced as a fit with the approach taken in other program components. “Encounters” were recognized as representations of real-life situations. Respondents who played generally believed that they could select options that corresponded with their intentions. Experienced consequences were acquaintance with meditation, concentration, and reflections on ideal and “healthy” selves, rumination, and adequate ways of responding.

I saw pretty quickly where they wanted to go with it. In that respect, it does well with what they do at Ciran.

Respondent #1

I have a computer, then it is no problem… I could use that game well… Later you find out: oh, it's not just a game. It is something to think about your own situation... Then you're not in the game, but you are in reality… All kinds of possibilities were offered (referring to response options in encounters): what I see as negative, in between, and what I see as 'good’… It was also a bit about … as I was during the illness... I could recognize myself in some situations: Yes, that was the old <patient name>… At the moment however, I react completely differently. I could see that. Maybe that also influenced me: my healing.

Respondent #3

During the game you were forced to concentrate; not wanting to go through it too fast… Actually you were just forced to use your concentration... It occurs to me that I ruminate long about something, and it also confronts you with that… Those first meditation exercises… I really needed to do it a few times ... That got me meditating…

Respondent #4

### Suggestions for Improvement

Respondents suggested to integrate LAKA, similar to other program elements patients normally adhere to. Additional support was desired by means of a personalized introduction early in the program, information about how to get something out of the game and about what is achieved afterward, professional feedback on situations in the game, and facilities for gaming at Ciran locations. Suggestions for playability were to match the pace of in-game interaction with skill or health status and to extend software support to multiple platforms.

I would certainly continue to offer it. Maybe someone should be designated to introduce it at an early stage... To show it, and to show what you can get out of it for yourself. In a playful way ... If an entire manual should be read, then you put it away quickly… Actually, I do most with the IPad. If that is possible... I rarely use a pc.

Respondent #4

I would let everyone play. I think if it is offered in <location name>... “If there is a psychologist… that is better. Immediately talk about those reactions taking place at that time… find out faster what kind of situations played a role in becoming so ill, and get better... Maybe you could combine it … a bit slower in the beginning and a little faster at the end.

Respondent #3

## Discussion

###  Principal Findings

This study primarily questioned why and by which patients with chronic pain and fatigue complaints is applied gaming for behavior change demanded during an IRP. Voluntary applied gaming (LAKA) was generally explained over time by perceived enjoyment and ease. Coping resources are important to solve delivery issues, get in control, and start using an applied game. Patient environment, health status, and performance expectancies were relevant factors for the usage of an applied game in conjunction with time and exposure.

Second, feasibility was described in preparation for a full-scale evaluation. A substantial number of patients played the game under noncommittal conditions. According to them, LAKA will be acceptable and useful. Active ingredients were recognized and deemed to be relevant in early stages of a rehabilitation process. Patients suggested delivering the game with social support through early and expeditious communication about how and when the game is relevant for their rehabilitation and with extended technical facilities.

### Strengths and Limitations

This study provides the first empirical results on a novel applied game for behavioral change in patients with chronic pain and fatigue complaints. Comprehensive information is presented on processes of self-selection, acceptance, and attrition, which provides rare insights into risk factors for bias in CBI evaluations [58]. Mixed methodology strategy worked well to triangulate QN findings with newly collected QL data. Important demand explanations are based on notable and robust statistical results supported by a decent sample size and clear illustrations with QL data. QN results that were not clearly illustrated with QL data, or were based on more selective patient samples, provided practical information and clues for future research. More than a final feasibility assessment, this study contributed with general and utilizable knowledge for the future deployment of applied gaming for FSS patients in practice.

Limitations should be considered when drawing general conclusions about the feasibility of applied gaming for FSS patients. Feasibility was not assessed against a control group level or a reasonable benchmark. Technology acceptance measures are commonly used, but they are often contextually adapted and serve in theory building rather than feasibility assessment. Furthermore, this study builds on pragmatic eligibility criteria and convenience sampling of Dutch patients. It cannot be ruled out that early judgments about the nature of the delivery mode affected results via self-selection. Performing a large number of explorative statistical tests threatens statistical power and internal validity. Caution should be exercised when interpreting causality in relationships between behavioral factors and behavioral intentions because independent and dependent variables were measured at the same point in time, and hedonic motivation was not clearly distinct from performance expectancies and behavioral intentions. Whether the use of technology acceptance questionnaires alone would be an appropriate method for assessing the usage of gaming technology, especially at a time when patients may have difficulty processing information, can be doubted and is not recommended. Finally, advanced statistical techniques such as partial least squares regression [59] or newer process analyses techniques [60] would have been appropriate, but were not used. QN method limitations were addressed by triangulation of key QN findings with QL data, comprehensive sample description, validity checks, residual analysis, and sensitivity analyses (for outlier removal, measures of association, and regression method; see Multimedia Appendix 7).

### Comparison With Prior Work

Researchers have stressed that a better understanding of the demand for CBIs is a major concern in overcoming barriers to treatment of patients with chronic pain and fatigue symptoms [2,3]. To our knowledge, this is the first empirical study on applied gaming for the delivery of behavioral intervention for patients with chronic somatic symptoms and functional problems. Findings suggest that voluntary engagement in applied gaming is strongly driven by positive affect. The importance of hedonic motivation for demand is remarkable because this is an often-omitted factor in previous research on the use of information technology in health care [27]. Concerns about utility, demonstrability of results, privacy, or consultation seemed to have a limited effect on demand in this case, when patients had no previous experiences. Ubiquitous interview quotes about “openness” hinted that inclinations to search for meaning or personal growth could partially explain demand for applied gaming [61,62]. Concluding that FSS patients will use an applied game “for the sake of the activity itself” is tentative. Applied gaming interventions are relatively new and barely institutionalized, and limited information was available to patients about the efficacy of LAKA or a similar game. It could also be that patients thought about usefulness and trustworthiness of care before deciding on following an IRP. Moreover, findings on influences of individual differences in coping styles and perceptions of control and ease on the usage of LAKA correspond with those of earlier studies that found a positive effect of internal locus of control on the adherence to a web-based positive psychology intervention [9]. Such results might also reflect differences in executive functioning or capacities for self-control [63].

Other remarkable QN results, which were not clearly illustrated with QL data, are discussed in connection with past research or as areas of future research. Findings on the effect of depressed mood on CBI usage have been heterogeneous [30]. This study pointed in the direction of a negative relationship, but found no statistically significant direct effect. This might be because of the comparatively high levels of psychopathologic symptoms of these FSS patients [64]. A moderation effect is indicated by extremely low BIs that were found exclusively in patients with low to neutral performance expectancies and high levels of depressive symptoms. Furthermore, computer anxiety and experience might explain differences in relationships between age and technology usage found in earlier studies [27]. Moreover, patients with lower scores for pain intensity and those who indicated fatigue as their primary complaint were more likely to self-select as a player, whereas patients with higher pain intensity played more once exposed. Further research on the usage of CBIs could focus on understanding “matches” between symptom characteristics, “readiness,” and demanded delivery mode of behavioral treatment for FSS populations [65]. Another research focus can be patient-environment interactions (ie, coping with issues at home, absenteeism, and return to work) as barriers and facilitators for demand within this target group. For informing design and implementation of computer- and game-based modalities, it is useful to proceed with qualitative research and by formulating and testing theoretically informed hypotheses on how usage varies by patient, program design, behavioral, and context factors [26,61,66-68].

The degree of implementation of LAKA for eligible patients is not satisfactory, as could be expected when a CBI is offered under ad libitum conditions [69]. Besides blending with face-to-face delivery and multiplatform distribution, solutions for additional support can be provided through Web-based features such as tailored messages, prompts, and support via email, chat, or message boards [4]. Acceptability and limited efficacy outcomes should be treated with caution but suggest that LAKA is potentially efficacious and sufficiently engaging to complete once or twice (2-4 hours). Moderately positive enjoyment by users may reflect that the design principle of LAKA was not entirely hedonic, maybe at the expense of “playability” aspects [70]. Eliciting reflective and meditative states, LAKA was pleasant for a patient with concentration problems; however, self-reflections seem to be at the expense of a more satisfactory speed of interaction. Moreover, the game appears to provide opportunity to realize ideal selves, which supports intrinsic motivation [57]. However, LAKA also triggered serious reflections about discrepancy with “actual” selves, which is associated with somatic symptoms and negative emotions [71]. “Slowness” was mentioned as a reason for disengagement, but self-awareness was not. One may also reflect about how self-awareness in virtual reality relates to bodily and behavioral representations of Avatars [72] because extremely low perceived knowledge improvement levels were exclusively reported by patients with high BMI levels at baseline. High-quality and adequately powered studies on the effects of LAKA and similar systems on functional domains are needed to clarify the roles of self-conscious and affective states, learning, and degree of engagement [16,17,73].

### Conclusion

Although these first empirical findings support that an applied game is used by FSS patients for enjoyment and convenience, it became very clear that many patients would not be reached with a behavioral intervention of this modality under voluntary conditions. Social factors remain highly important for reaching many patients. LAKA will be feasible as a short and early intervention for patients, with adjustments of social and technical support. A next step in deployment and evaluation of the efficacy and cost-effectiveness of LAKA in a controlled study is recommendable.

## Appendix

Multimedia Appendix 1 Conceptual framework.

Multimedia Appendix 2 Serious gaming page.

Multimedia Appendix 3 LAKA details.

Multimedia Appendix 4 All measurement details.

Multimedia Appendix 5 Regression models specified.

Multimedia Appendix 6 Interview schedule.

Multimedia Appendix 7 Method sensitivity analyses.

## Abbreviations

BI: behavioral intention
CBI: computer-based interventions
FSS: functional somatic syndromes
IRP: interdisciplinary rehabilitation program
QL: qualitative
QN: quantitative
