# Supplementary material for: Feasibility of Applied Gaming During Interdisciplinary Rehabilitation for Patients With Complex Chronic Pain and Fatigue Complaints: A Mixed-Methods Study
Source: JMIR Serious Games. 2016 Apr 1;4(1):e2. doi: 10.2196/games.5088 (PMC4833876; doi:10.2196/games.5088)
Supplement: Multimedia Appendix 1 [file games_v4i1e2_app1.pdf]

## Multimedia Appendix 1: [Conceptual framework]

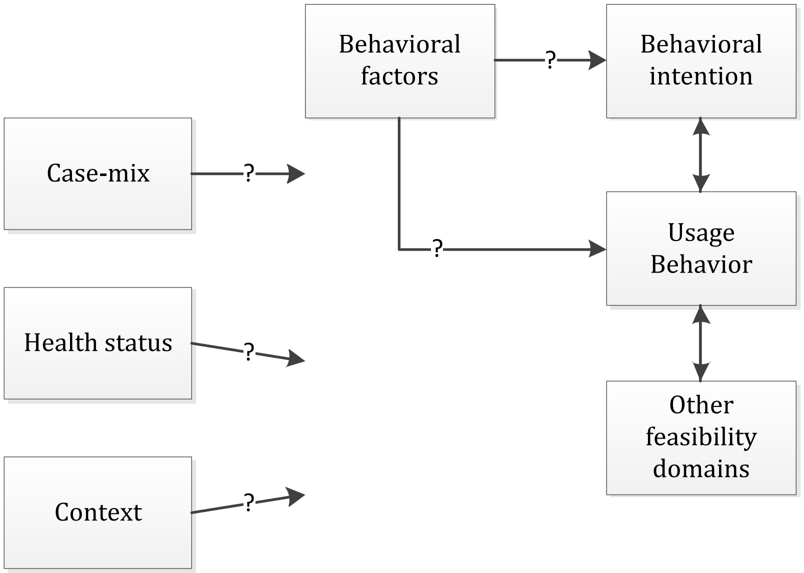


| **Definition of feasibility domain [25]** | **Operationalization** |
|---|---|
| **(1st) Demand:** The estimated or actual use of selected intervention activities in a defined intervention population or setting. | Behavioral intention (BI); the degree to which a person has formulated conscious plans to use the system determined by behavioral factors, and; usage behavior (frequency, length, and amount). |
| **(2nd) Acceptability:** How the intended individual recipients, both targeted individuals *and those involved in implementing programs[a],* reacted to the intervention. | Patient or end user perceived appropriateness. Criteria for appropriateness are 1) intrinsically motivating/perceived enjoyment 2) user friendly/ease of use, and 3) perceived knowledge improvement. |
| **Implementation:** The extent, likelihood, and manner in which an intervention can be fully implemented and evaluated as planned and proposed. | Degree of execution: assessment of study attrition and usage attrition by the rates of survey submission and program usage. |
| **Practicality:** Can an idea, program, process, or measure be carried out with intended participants using existing means, resources, and circumstances and without outside intervention? | The apparent ability of participants to carry out intervention activities evidenced by the presence of a successful log-in. The extent to which the system is used by those who possess gaming platforms. (How) playing LAKA was fitted into the day- and treatment schedule of patients. |
| **Limited efficacy:** Does a new idea, program, process, or measure show promise of being successful with the intended population? | Intended and perceived effects of system usage on knowledge and the process of rehabilitation. |

[a] Not addressed in this study
