# Supplementary material for: Feasibility of Applied Gaming During Interdisciplinary Rehabilitation for Patients With Complex Chronic Pain and Fatigue Complaints: A Mixed-Methods Study
Source: JMIR Serious Games. 2016 Apr 1;4(1):e2. doi: 10.2196/games.5088 (PMC4833876; doi:10.2196/games.5088)

Multimedia Appendix 2: [Serious gaming page]

This serious gaming web-page was incorporated in a web-portal (secured with an identification number and self-chosen password) that is used by patients to inspect their treatment schedules and treatment records. The serious gaming page guided visitors through procedures for informed consent, survey submission, resources for downloading and installation, and contact information for questions and issues. Questionnaire submission enabled access to the game software, which was also provided automatically if patients refused, or did not comply with study procedures in time. Information on the web page informed that LAKA focuses on positive health domains rather than symptom or risk reduction, and that it would be interesting to play more than once. Questions and issues were administrated and solved by process managers and ICT specialists at the headquarters of Ciran.

Three screenshot examples are provided (Dutch):

a) Main menu
b) Informed consent
c) Introduction of Laka and access to the first additional questionnaire

a) Main menu

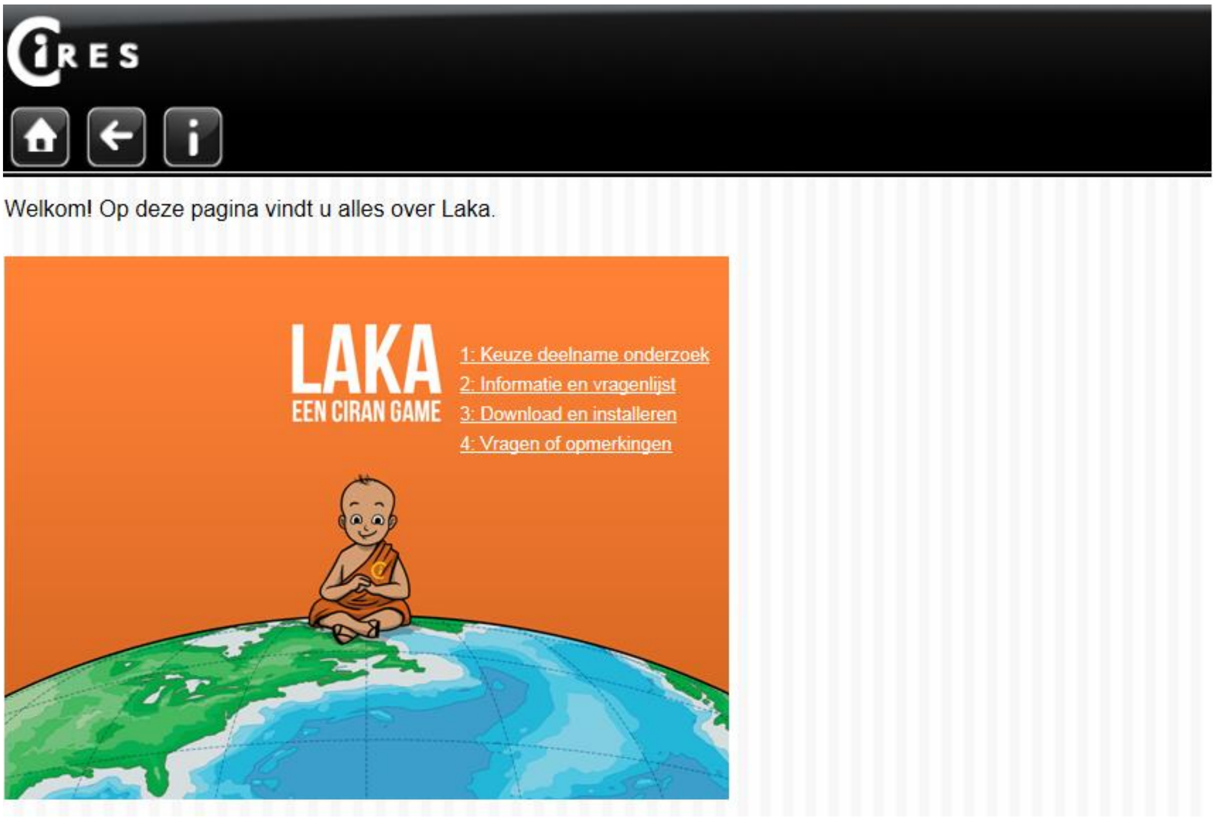

1: Informed consent, 2) Information (LAKA) and questionnaire, 3) Download and installation, 4) Questions or remarks

b) Informed consent

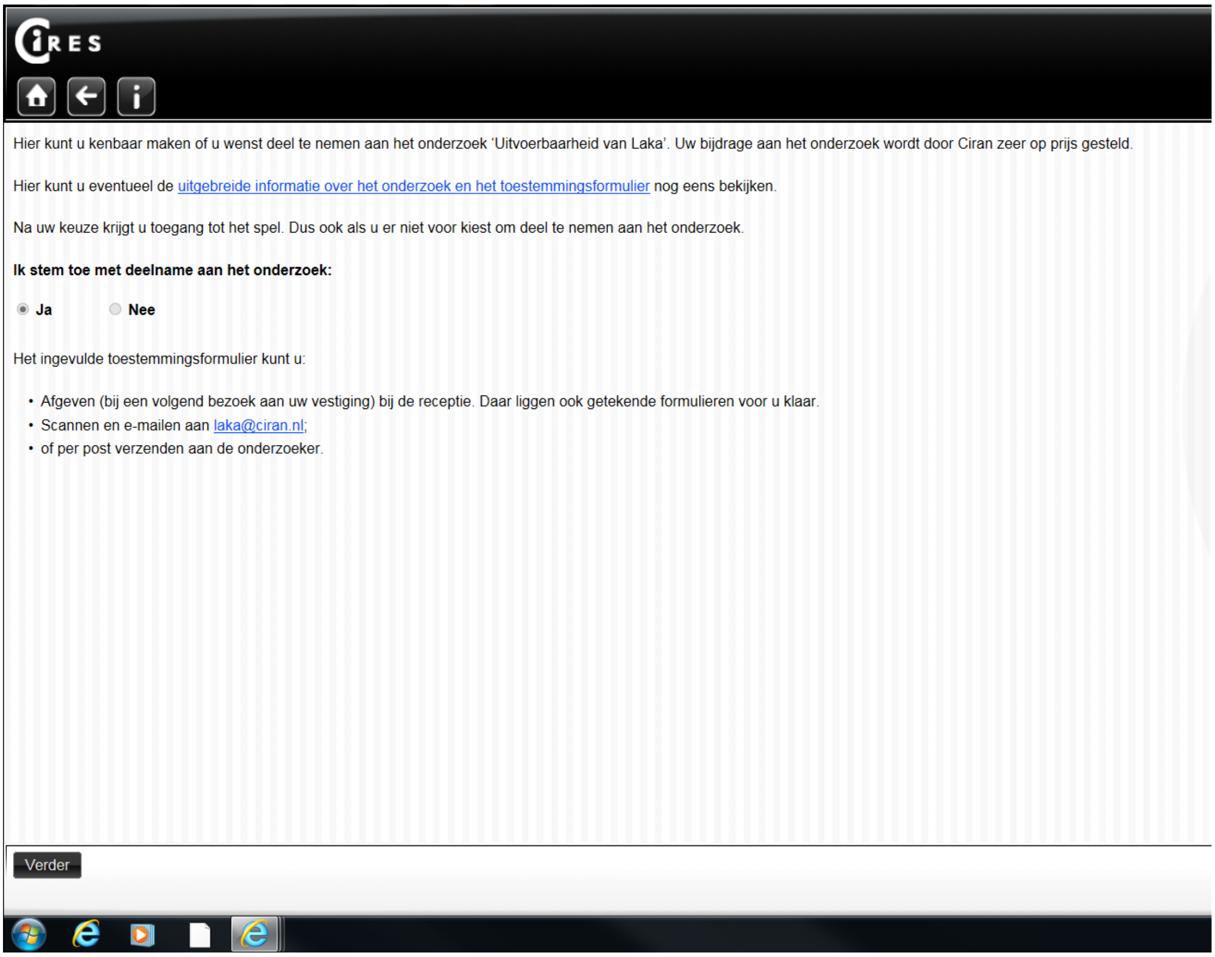

CIRES

Hier kunt u kenbaar maken of u wenst deel te nemen aan het onderzoek 'Uitvoerbaarheid van Laka'. Uw bijdrage aan het onderzoek wordt door Ciran zeer op prijs gesteld.

Hier kunt u eventueel de uitgebreide informatie over het onderzoek en het toestemmingsformulier nog eens bekijken.

Na uw keuze krijgt u toegang tot het spel. Dus ook als u er niet voor kiest om deel te nemen aan het onderzoek.

Ik stem toe met deelname aan het onderzoek:

Ja **Nee**

Het ingevulde toestemmingsformulier kunt u:

- Afgeven (bij een volgend bezoek aan uw vestiging) bij de receptie. Daar liggen ook getekende formulieren voor u klaar.
- Scannen en e-mailen aan laka@ciran.nl;
- of per post verzenden aan de onderzoeker.

Verder

c) Introduction and first questionnaire (t1)

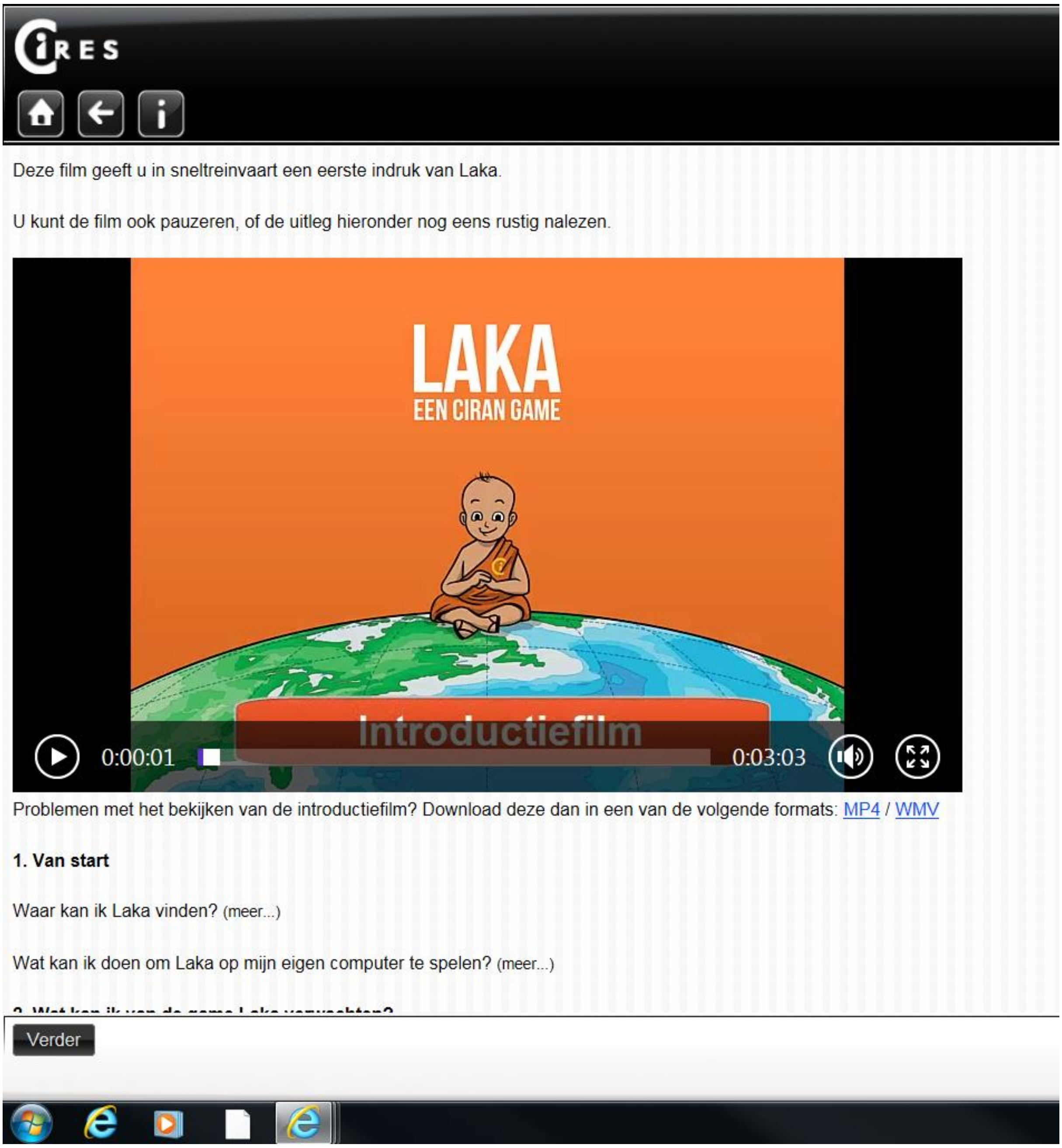

Supplement: Multimedia Appendix 2 [file games_v4i1e2_app2.pdf]
