# Supplementary material for: Feasibility of Applied Gaming During Interdisciplinary Rehabilitation for Patients With Complex Chronic Pain and Fatigue Complaints: A Mixed-Methods Study
Source: JMIR Serious Games. 2016 Apr 1;4(1):e2. doi: 10.2196/games.5088 (PMC4833876; doi:10.2196/games.5088)
Supplement: Multimedia Appendix 3 [file games_v4i1e2_app3.pdf]

Multimedia Appendix 3: [LAKA details]

Functional requirements and links with module component

Module components	Expert playability/usability information
**Introduction**: Select Avatar (name), introduction of story and LAKA	Patients might like to choose their Avatars' appearance beyond a ('typical' 45 year old Caucasian) male or female.<br>Lengthy story introduction → Substantially reduced to prevent boredom. Conversations with LAKA can be skipped if game is played for the second time. Level of interactivity is limited, but not unpleasantly. LAKA character is 'funny'.
**Encounters:**<br>(see textbox 3, screenshot b and c)	Interface works intuitively.<br>5 options for responding general provides a sense of autonomy, but sometimes players experience that *their own* implementation intentions are not well represented by (one of the) given options.
**Mood scenarios**:<br>Avatar copes with a stressful situation	Modes of presenting text messages on screen ('hold on to the emotions of your Avatar') was adapted several times for the sake of clarity and having enough time to read.
**Tours**: Pictures with 'tour-guide' voiceovers inform about the location visited by the Avatar	Players are inclined to skip: switching from immersive simulation environments to listening to guided tours seems to require mental effort Players may avoid breaking immersion. → Tours can be skipped even more easily (one 'click').
**Loading screen**:<br>Maps of Avatar locations.	Generally liked
**Mini-games:**<br>Plumber (puzzle) and race games	Puzzle tasks are a bit difficult → players automatically proceed after 3 fails.
**Reflections**:<br>Patients provide self-assessment of …, and receive feedback on ... the quality of their responses. (screenshot d)	Feedback is abstract and general (not instantly on specific choices). There are no 'auditory' explanations of targeted behavioral qualities (goals), and (vicarious) consequences of behavior are unpredictable. On the one hand, players are free to affirm what they value personally. On the other hand, players might like clearer goals.
**Attention training:**<br>Guided focused attention and open awareness meditation exercises	Although there may be similarities between ideal mental states in gaming (immersion) and those of (various) meditation practices, (switches to) 'low stimulus' contexts in attention trainings in contrast with other ('high stimulus') module components may be demanding for players.
**Overall (all above)**	The game play is calm, may be challenging due to switches between immersive and reflective states. It is expected to be attractive, immersive, and interesting enough to be played one, or maybe 2 times until interest is lost.

Screenshots and trailers

a) Opening screen

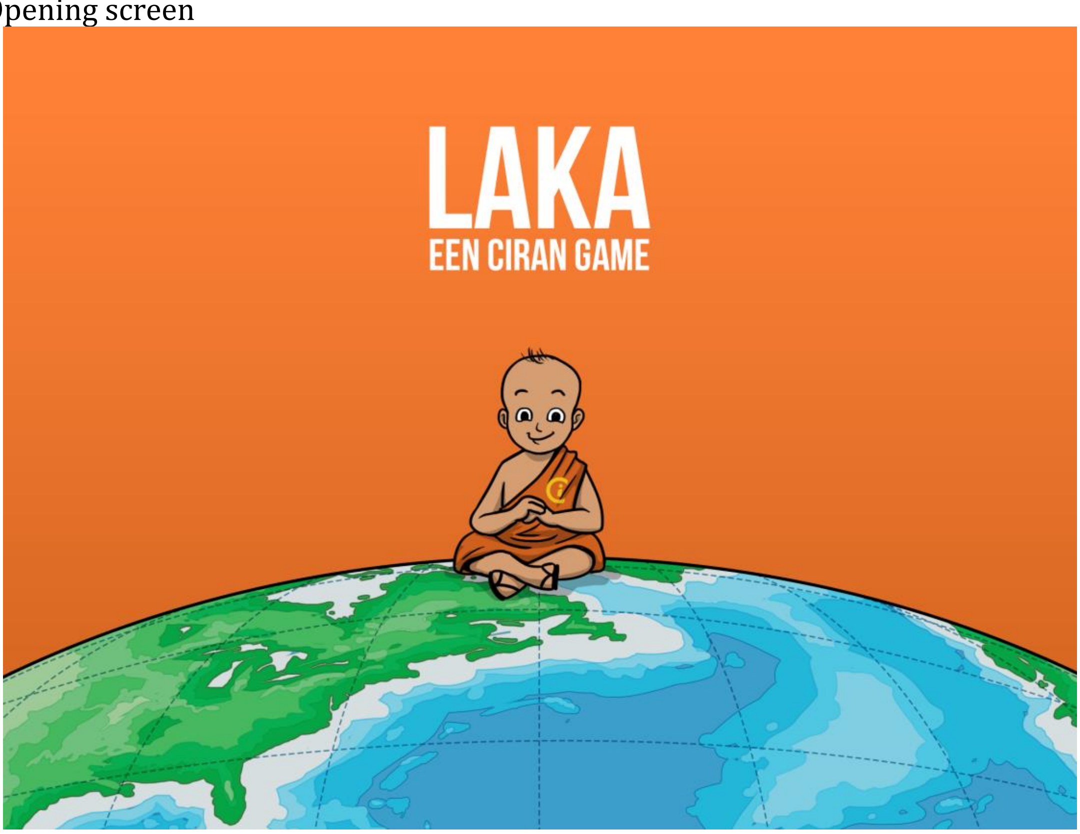

b) London Hyde Park

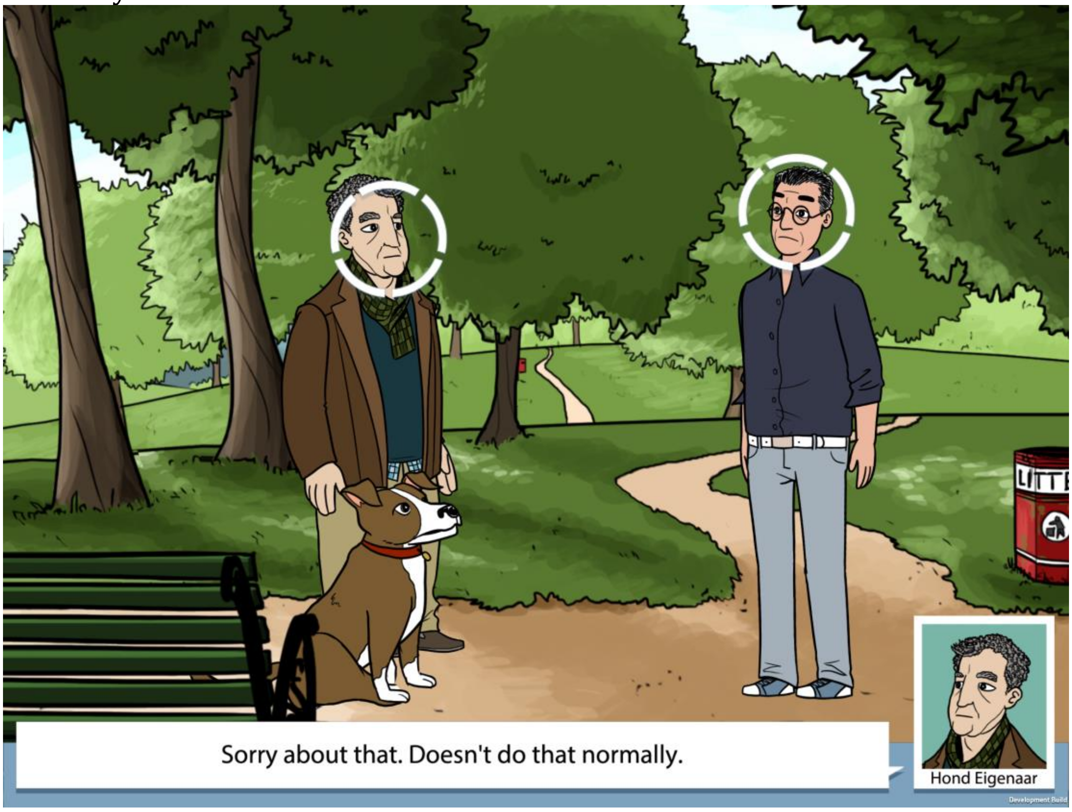

c) Asia Lhasa

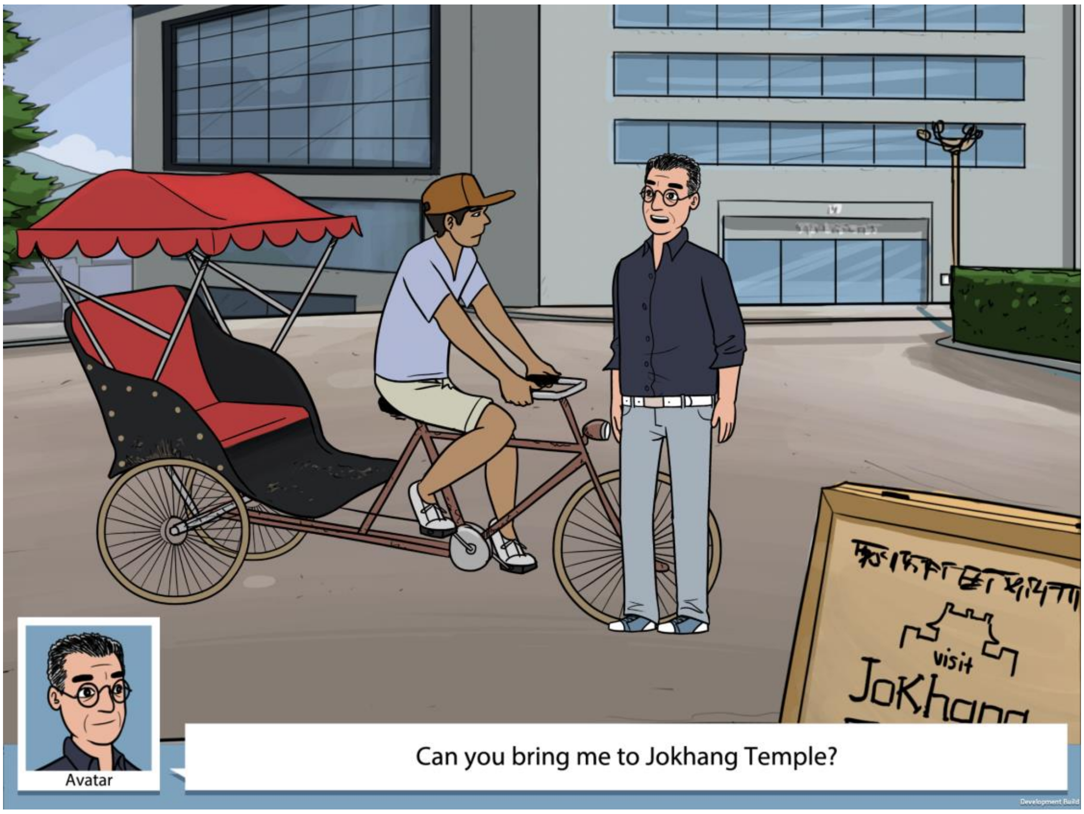

d) Puzzle screen

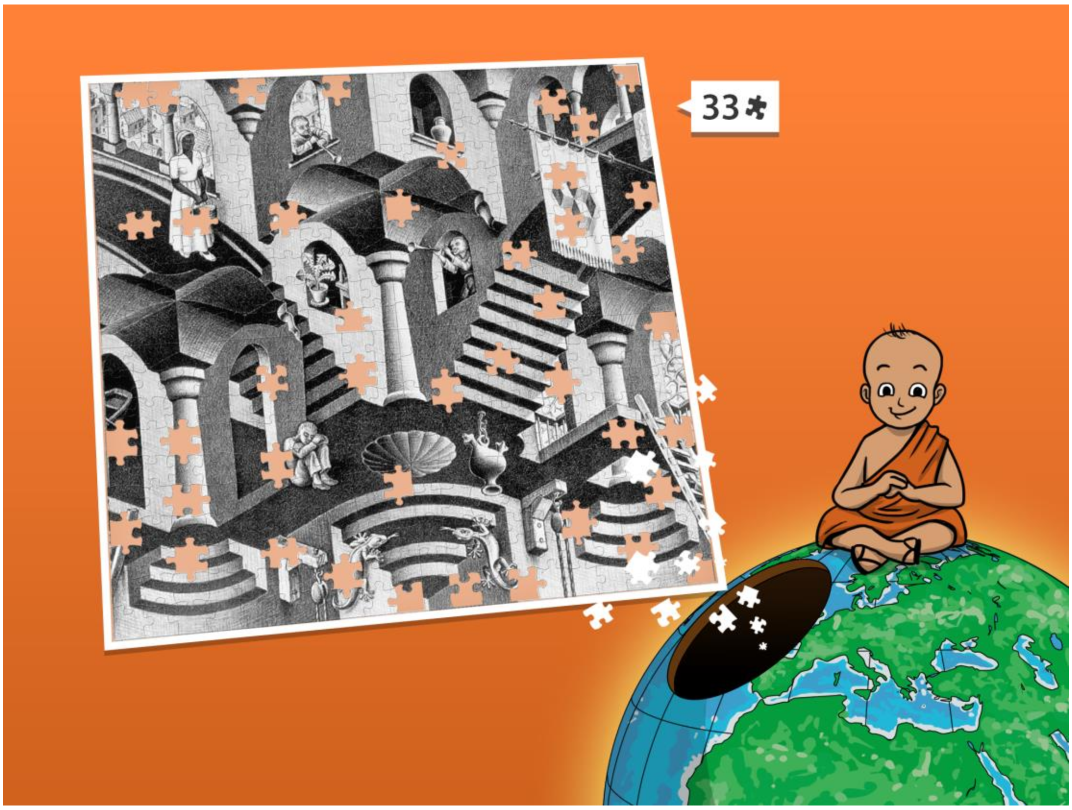

e) Protagonist prototypes

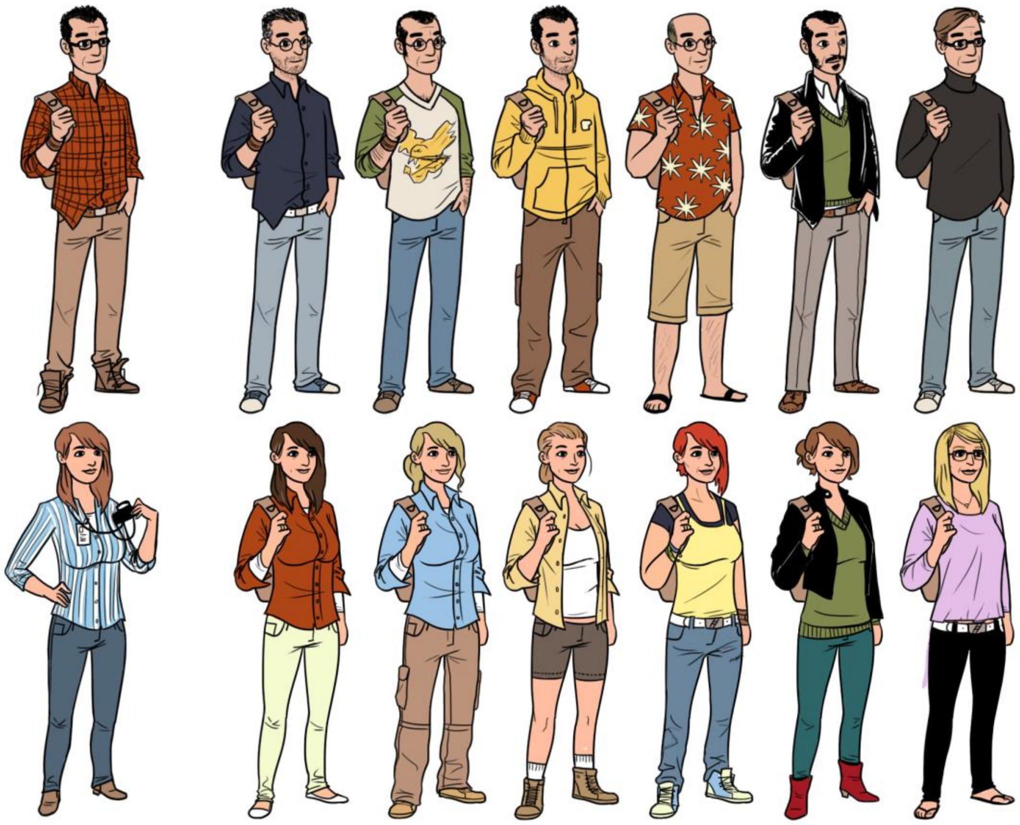

f) Laka prototypes

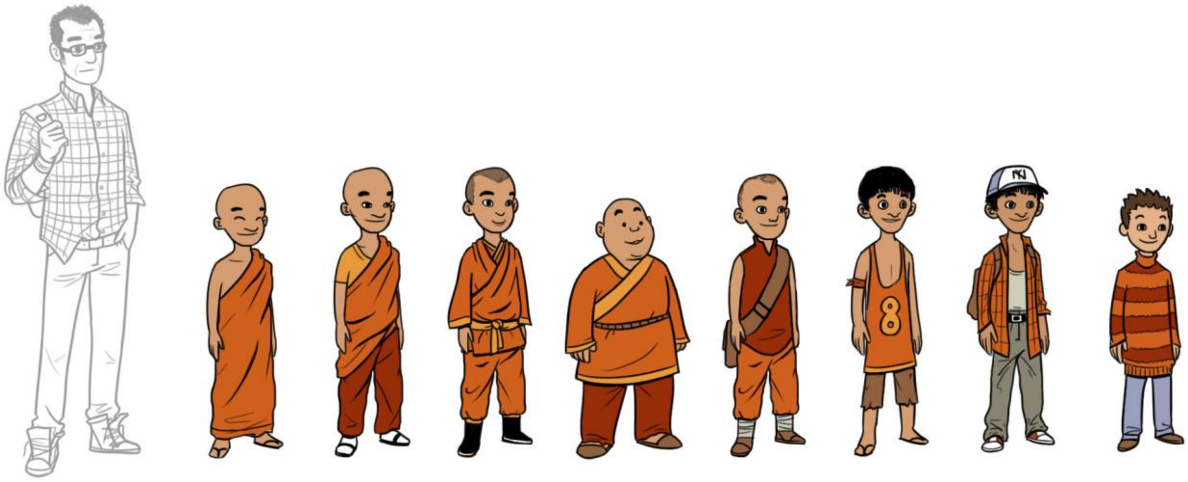

Trailer of the experimental version of the game (Dutch)
http://www.ciran.nl/laka/lakaNed.php

Prototype trailer (English)
http://www.ciran.nl/laka/lakaEnglish.php
