# Supplementary material for: Feasibility of Applied Gaming During Interdisciplinary Rehabilitation for Patients With Complex Chronic Pain and Fatigue Complaints: A Mixed-Methods Study
Source: JMIR Serious Games. 2016 Apr 1;4(1):e2. doi: 10.2196/games.5088 (PMC4833876; doi:10.2196/games.5088)
Supplement: Multimedia Appendix 4 [file games_v4i1e2_app4.pdf]

Multimedia Appendix 4: [All measurement details]

All patients eligible for treatment gave approval to process their records for scientific research. The 2 additional surveys for this study were implemented in the same password-protected web-survey system that patients had already used for their routine baseline assessments. All QN data were stored at the rehabilitation center and transferred in anonymous form to the researchers before processing. All scales were checked for internal consistency. Scales were omitted for further analysis if Cronbach's alpha was lower than .7, and treated with caution if alpha was between .7 and .8 [39].

This appendix provides additional information about:

1. Additional technology acceptance measures p. 1-3
2. Routine baseline web-survey measures p. 4-5
3. Screen-shot example of a web-survey page p. 6
4. Factor analysis behavioral factors and intention at t1 p.7

1) Technology acceptance measurement

- A convenience sample was used, because attrition rates needed to be better understood. Nevertheless, a power analysis was performed in advance, based on the estimation that 400 (actually 410) patients would start their treatment during a 3 month inflow period. A minimum of 100, and a maximum of 200 participants were expected to participate (actually 116).
- Before consent, participants were informed that completion of the two additional surveys would take less than ten minutes each.
- Patients received a reminder per e-mail after two weeks, and in a scripted telephone call after three weeks.
- Dutch item formulations were based on back-to-back translations of a study that verified measurement validity across cultures and languages [45].
- Item formulations were adjusted for pre- and post-usage assessment [24].
- By exception, scales for technology acceptance modeling were visible on the computer screen as groups of items in accordance with previous recommendations [46].
- The second survey automatically adapted to the usage behavior of respondents. All participants received the intention to use scale at t2. However, the remaining scales were only to be filled out by those respondents who, at least, completed the first module of the game.
- The following textbox provides further information about scales that were used:

Actual usage behavior
- Definition: Actual interaction with the applied game LAKA.
- Variables:
  - Frequency (or number of 'session days') was determined by counting the number of dates on which progress in game-play was registered.
  - Length of exposure (or total amount of time spend on game-play) was calculated by summing the lengths of time-intervals between log-ins and subsequent logs of in-game decisions made by participants.
  - Progress in LAKA was determined as the number of completed encounters.

Behavioral intention [24]
- Definition: The degree to which a person has formulated conscious plans to perform, or not perform, some specified future behavior.
- Item example (1/3 items) and scale: I intend to use … in the next four months. All technology acceptance variables were calculated as scale item means.
- Cronbach α at t1: .946 (N:116); at t2: .970 (N=93)

Performance expectancy/ perceived usefulness [24]

- Definition: The degree to which an individual believes that using the system will help him or her to attain gains in task performance.
- Argument for inclusion in explorative model: Deemed indispensable for modeling health information technology demand [39].
- Item example (1/3 items): Using LAKA (would) enable(s) me to accomplish my personal health goals more quickly.
- Cronbach α at t1: .95 (N=116); at t2: .91 (N=34)

Effort expectancy/ perceived ease of use [24]
- Definition: The degree of ease associated with the use of the system.
- Argument for inclusion: Deemed indispensable for modeling health information technology demand [41].
- Item example (1/3 items): Learning how to use LAKA is/was easy for me.
- Cronbach α at t1: .92 (N=116); at t2: .94 (N=34)

Social influence [24]
- Definition: The degree to which an individual perceives that important others believe that he or she should use the new system.
- Argument for inclusion: To explore the role of perceived normative pressure, an important factor in the Unified Theory of Acceptance and Usage of Technology (UTAUT) and theory of planned behavior [37].
- Item example (1/3 items): People who are important to me think that I should use LAKA.
- Cronbach α at t1: .96 (N=116)

Facilitative conditions/ Perceived behavioral control [24]
- Definition: The degree to which an individual believes that an organizational and technical infrastructure exists to support use of the system.
- Argument for inclusion: To explore the importance of perceived external control, an important factor in the unified theory of acceptance and usage of technology [37]. Potential target for intervention support.
- Item example (1/2 items): I would have the resources necessary to use LAKA.
- Cronbach α at t1: .76 (N=115)

Computer self-efficacy [37]
- Definition: The degree to which an individual beliefs that he or she has the ability to perform specific task/job using computer.
- Argument for inclusion: Potential target for (technical) support.
- Item example (1/2 items): I could complete a task using the system...
- …, if I had just the built-in help facility for assistance.
- Cronbach α at t1: .70[a] (N=115)
- Remarks: Before scoring the items, a task definition (completion of a module or destination) was given in the survey. The internal consistency of this scale was, unlike other scales, only just sufficient. This might be due to the somewhat more complex manner of questioning. Results based on this measure should be treated with some extra caution.

Computer anxiety [37]
- Definition: The degree of an individual's apprehension, or even fear, when she/he is faced with the possibility of using computers.
- Argument for inclusion: Potential target for (emotional) support.
- Item example (1/3 items): I hesitate to use LAKA for fear of making mistakes I cannot correct.
- Cronbach α at t1: .87 (N=115)

Hedonic motivation/ Perceived enjoyment [23]
- Definition: The fun or pleasure derived from using a technology.
- Argument for inclusion: Technologies with a self-fulfilling rather than instrumental value for users, such as virtualization and consumer technologies, may constitute a boundary condition for (early) technology acceptance models [41]. Consequently, better predictions may be achieved with complementary affective and automatic components, including hedonic motivation and habit [23].
- Item example (1/3 items): Using LAKA would be fun.

- Cronbach α at t1: .93 (N=116); at t2 .93 (N=35)

Habit
- Definition: The extent to which people tend to perform behaviors automatically because of learning (from past behavior) [23].
- Argument for inclusion: See argumentation above.
- Operationalization: The sense of familiarity characteristic of a habit was assumed present if participants reported 1) computer game play in the past month, and 2) a minimum frequency of monthly computer game play, and 3) internet usage on 6-7 week days.
- Questionnaire items:
  - How much time has been passed since the last time you played a computer game (Never played a computer game before, > year ago, < year ago, < month ago, or < week ago)?
  - How frequently did you play computer games (on average) within the past year (yearly, monthly, weekly, daily)?
  - How frequently did you use the internet (on average) within the past year (< once per month, … on 6-7 week days)?
  - Please check with all game genres you have experience with (more than 10 hours of game-play) (Adventure, i.e. Super Mario, role-play games, i.e. …., etc.)?

Trust [24]
- Definition: 'A contextual belief to reflect users' increased concerns about privacy and security when using emerging technologies in contexts that require online transmission of personal and sensitive information'.
- Argument for inclusion: Trust may have a high relevance in health care contexts where privacy and result demonstrability are important, and can reflect reputations of technology providers [39].
- Item example (1/3): LAKA would provide access to sincere and genuine rehabilitation care services.
- Cronbach α at t1: .86 (N=115)

Remarks: Explorative factor analysis revealed a logical and workable pattern of item inter-correlations.

Perceived knowledge improvement [44]
Four items of the perceived knowledge improvement scale (with the highest factor-loadings) were adopted from the EGameFlow questionnaire for serious game assessment [50], and translated in Dutch (for example: "The game increases my knowledge"). A strong internal consistency (Cronbach Alpha=.97, N=32) and strong correlation with post-usage perceived usefulness (ρ=.80, *P* <.001) provide support for validity.

2) Baseline measures

- Patients without convenient access to a personal computer were familiar with opportunities to fill-out web-surveys at local facilities.
- The survey system enabled to fill out questionnaires in a preferred order, self-paced, and at several occasions.
- By standard, questionnaire items were presented one by one on the computer screen. Responses were checked for completeness, and could be reviewed and changed.
- Results of internal consistency checks of previously validated questionnaires are provided in the following table:

Questionnaire	(Sub)-scales	Cronbach alpha (N, items)
Utrecht Coping List (UCL)[a]	Active engagement	.82 (410, 7)
	Seeking social support	.89 (410, 6)
	Passive/depressive response pattern	.73 (410, 7)
	Palliative response*dropped*	.66 (410, 8)
	Avoidance*dropped*	.62 (410, 8)
	Expression of emotions*dropped*	.64 (410, 3)
	Comforting thought	.70 (410, 10)
Symptom Checklist (SCL-90)[b]	Psychopathology	.97 (408, 90)
	Depression	.90 (408, 16)
	Anxiety	.87 (408, 10)
	Somatic Symptoms	.81 (408, 12)
	Agoraphobia	.87 (408, 7)
	Insufficiency of thinking and acting	.85 (408, 9)
	Distrust and interpersonal sensitivity	.91 (408, 17)
	Hostility	.83 (408, 6)
	Sleeping problems	.79 (408, 3)
Pain coping and cognition (PCCL)[c]	Pain coping	.80 (353, 11)
	Externalizing*dropped*	.69 (353, 8)
	Internalizing	.79 (353, 11)
	Catastrophizing	.83 (353, 12)
Tampa Scale of Kinesiophobia (TSK)[d]	Kinesiophobia	.79 (352, 17)
Checklist Individual Strength (CIS)[e]	Total CIS	.90 (402, 20)
	Subjective fatigue	.85 (402, 8)
	Motivation (problem)	.81(402, 4)
	Concentration (problem)	.90(402, 5)
	Physical (in)activity	.87 (402, 3)
Utrecht Burnout Scale (UBOS-a)[f]	Mental exhaustion	.88 (406, 5)
	Competence	.82 (405, 6)
	Distancing	.82 (405, 4)

[a] UCL: On 4-point Likert-scale items UCL patients rated how frequently they coped with stress in problem focused ways like active engagement, fostering reassuring thought, or seeking distraction, versus emotion focused ways like passive coping, avoidance, or expression of emotions. A strategy of seeking social support co-varies with both dimensions [38].
[b] SCL: SCL-90 is a multi-dimensional psychopathology indicator. With 90 (5-point Likert) items patients rated the extent to which they recently suffered from various psychological and physical symptoms [49].
[c] PCCL: With 42 items, rated on 6-point Likert scales, PCCL assesses self-reported usage of various pain coping strategies, perceived internal and external pain control, and negative thinking about catastrophic consequences of pain [50]. PCCL was only taken by patients reporting mild to severe pain intensity (NRS).
[d] TSK: The Tampa scale for kinesiophobia (filled in by patients who indicated the presence of mild to severe pain on a numerical rating scale: N=352) was developed and validated for patients with low back pain or fibromyalgia [51]. With 4-point Likert scale items it indicates the degree of pain-related fear, or whether activity level is influenced by fear of injury with avoidance behavior as a result.
[e] CIS: The Checklist Individual Strength measures subjective fatigue and associated behavioral aspects (concentration and motivation problems). Patients report, with 7-point Likert scales, the extent to which they agree with statements about one's feelings over the last two weeks.

[f] UBOS-a: The Dutch version of the Maslach Burnout Inventory, called UBOS-a (Utrecht Burnout Scale), indicates level of burn-out, and distinguishes employees with burnout complaints from healthy workers and from those with other psychiatric symptoms. Subscale scores were determined as the mean of subscale (7-point Likert) item scores. Presence of burnout was determined according to the "exhaustion + 1" criterion for research in non-clinical populations: (very) high scores for exhaustion and mental distance combined with a (very) low score on competency [43].

Further measurement details are presented in following textbox:

- An **environmental issue** was deemed present if patients perceived to have a problem with family, an unstable living situation, or to be going through a major life event.
- Four dichotomous variables indicated **treatment facility** (A, B, C, or D).
- Patients indicated the **course of their complaints** by whether their symptoms were recurrent, had been stabilizing, deteriorating, or improving over time and **duration**: for how long their current symptoms had been lasting (0-3 months, 3-6 months, 6-12 months, 1-2 years, or more than 2 years).
- Work participation is objectively indicated by (self-) **employment status** (yes or no), and number of **weekly work hours** (according to employment contract).
- **Absenteeism** (due to symptoms) was reported: yes (1) vs. no (0). If patients were absent, they indicated whether there absence was **partial or complete**, and for **how long they had been sick listed** (since day:month:year).
- Patients rated on a 5-point Likert scale whether they experienced their present **general health** as poor, fair, good, very good, or excellent (How do you generally think about your health status?"). Formulation of this item largely corresponds with general health items included in various Health related Quality of Life instruments.
- **Perceived distance to previous role participation** due to somatic symptoms were assessed with several 11-point numerical rating scales. These were introduced as follows: "The following question is about" …
    - "… work life"… "In your case, we mean (employment / own business / household work / active participation in the activities aimed at the return to the labor market)"….
    - "… non-work life." … "Think of activities as self-care (washing, dressing), to walk, not being tied to home, family and leisure activity, travel, sports and club activity, study."….

  Then patients were asked: "Indicate (with the following slide) how far you are removed from your former level of functioning in your (non-) working life before the start of your current complaint?" (2-items). The internal consistancy (Crohnbach alpha) of this 2-item scale was .702 (N=410)

3) Web-survey page example ('Intention to use' scale, from the survey at t1):

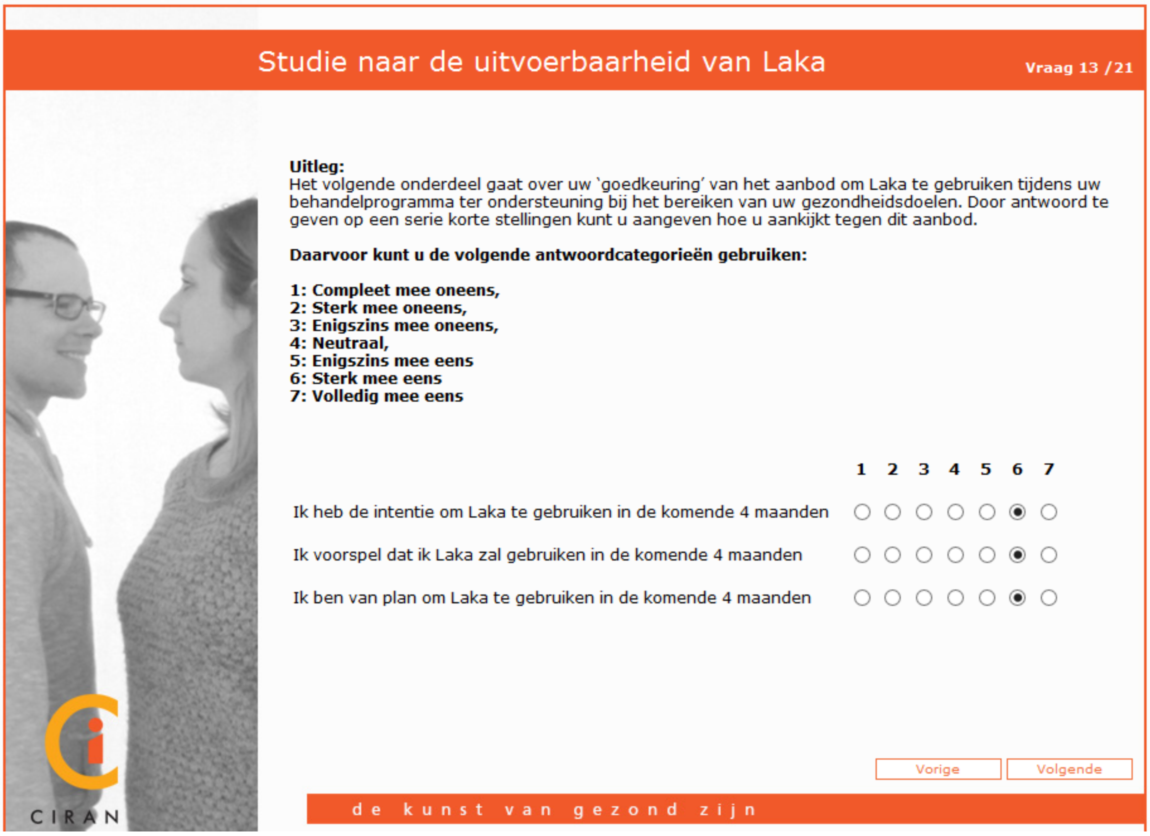

Studie naar de uitvoerbaarheid van Laka

Vraag 13 /21

Uitleg:
Het volgende onderdeel gaat over uw `goedkeuring' van het aanbod om Laka te gebruiken tijdens uw behandelprogramma ter ondersteuning bij het bereiken van uw gezondheidsdoelen. Door antwoord te geven op een serie korte stellingen kunt u aangeven hoe u aankijkt tegen dit aanbod.

Daarvoor kunt u de volgende antwoordcategorieën gebruiken:

1: Compleet mee oneens,
2: Sterk mee oneens,
3: Enigszins mee oneens,
4: Neutraal,
5: Enigszins mee eens
6: Sterk mee eens
7: Volledig mee eens

	1	2	3	4	5	6	7
Ik heb de intentie om Laka te gebruiken in de komende 4 maanden	○	○	○	○	○	◉	○
Ik voorspel dat ik Laka zal gebruiken in de komende 4 maanden	○	○	○	○	○	◉	○
Ik ben van plan om Laka te gebruiken in de komende 4 maanden	○	○	○	○	○	◉	○

Vorige | Volgende

4) Factor analysis (PCA): Pattern matrix of Oblimin rotated factor solution (with Kaizer Normalisation)

	Component							
	1	2	3	4	5	6	7	8
PU4	,947							
PU1	,943							
PU3	,892							
PU2	,608							
CA3								
CA5		,840						
CA2		,830						
CA1		,814						
PBC2		-,591						
CSE3[a]		,473					,437	
CA4		,472		-,435				
PBC3		-,460						
CSE4		,452						
SI4			,966					
SI2			,955					
SI3			,937					
BI2				,874				
BI3				,866				
BI1				,795				
CSE2					,845			
CSE1					,727			
PEOU3						,882		
PEOU1						,875		
PEOU2						,834		
**HM2[b]**						**,413**		
**HM3**								
**HM1**								
PBC1							,883	
SI1								-,729
TR3								-,612
TR2								-,592
TR1								-,547

[a] Items excluded in scales

[b] Hedonic motivation was not clearly conceptually distinct from other scales and overlapped with performance and effort expectancies.
