# Supplementary material for: Feasibility of Applied Gaming During Interdisciplinary Rehabilitation for Patients With Complex Chronic Pain and Fatigue Complaints: A Mixed-Methods Study
Source: JMIR Serious Games. 2016 Apr 1;4(1):e2. doi: 10.2196/games.5088 (PMC4833876; doi:10.2196/games.5088)
Supplement: Multimedia Appendix 5 [file games_v4i1e2_app5.pdf]

Multimedia Appendix 5. [Specified regression models]

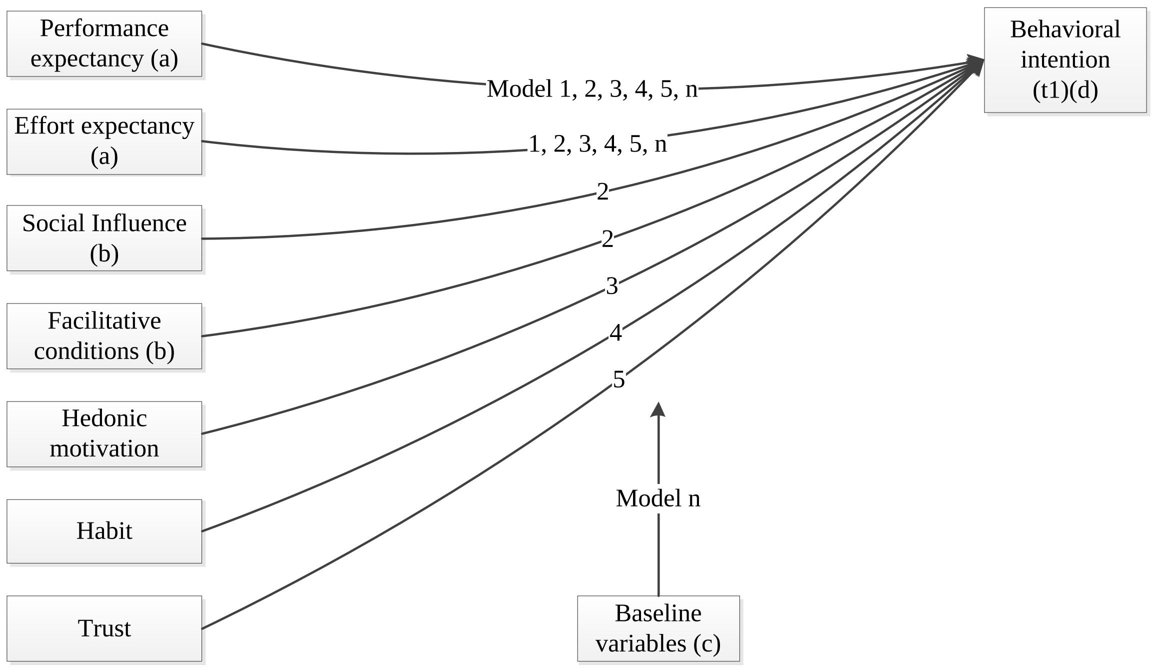

[a] Performance and effort expectancies specified a basic regression model. Explorative modeling encompassed the addition of behavioral predictors to this basic model.
[b] Social influence and perceived behavioral control were added to test direct predictors of the Unified Theory of Acceptance and Use of Technology [37]
[c] Interaction terms were tested for statistical significance in addition to the basic factors, if a bivariate scatterplot revealed obviously divergent directions or slopes of linear regression lines for sub-groups marked by dummy coded moderator variables: female, 45 years or older, low education, high education, environmental issue, treatment facility A-D, employment status, part time work, absenteeism, full absenteeism, > 6 months absenteeism, moderate or severe pain, primarily fatigue, primarily pain, primarily musculoskeletal disease, burnout, high anxiety, high somatization, high depression, CIS physical activity < mean (Chronic Fatigue Syndrome population), CIS fatigue severity < mean CFS, CIS concentration problems < mean CFS, CIS motivation problems < mean CFS, medication usage, previous specialized treatment, above average active engagement, overweight, symptom deterioration, symptom recurrence, and > 2 years symptom duration). Interaction models were build-up of centered variables to prevent multi-collinearity.
[d] Time-point 1: before usage.
