# Supplementary material for: Feasibility of Applied Gaming During Interdisciplinary Rehabilitation for Patients With Complex Chronic Pain and Fatigue Complaints: A Mixed-Methods Study
Source: JMIR Serious Games. 2016 Apr 1;4(1):e2. doi: 10.2196/games.5088 (PMC4833876; doi:10.2196/games.5088)
Supplement: Multimedia Appendix 6 [file games_v4i1e2_app6.pdf]

**Multi-media appendix 7: [Interview schedule]**

### *Introduction*

The interview began with a short (scripted) introduction.

### *Open questions*

Open vragen in het Nederlands (Open questions in English):
*Eerste reactie (Initial response)*

- Wat vond u van het aanbod om een videogame te spelen? (How did you think about the offer to play a computer game?)
- Waarom heeft u daar toen wel/geen gebruik van gemaakt? (Why did you / did not make use of it?)

*Gebruikerservaring: Indien gebruik hoog, ga naar oneven, indien gebruik laag, ga naar even nummers. Evt korte demonstratie van onderdelen van Laka als geheugensteun. (User experience: If high usage, go to uneven if low use, go to even numbers. Possibly short demonstration parts of Laka as a reminder)*

1) Wat was uw reactie toen u het speelde? (What was your reaction when you played it?)
2) Hoe kwam het dat u er niet aan bent begonnen? (How come you did not start?)
3) Hoe kwam het dat u stopte/doorging? (How come you stopped/proceeded?)
4) Wat vindt u ervan? Wat was goed/slecht? (What did you think of it? What was good/bad?)

*Tijdens het programma is er voor u wellicht iets veranderd… (During the program, some things may have changed for you...)*

- Kunt u vertellen hoe u dit beleefd heeft? Wat is u het meeste bijgebleven? (Can you tell how you experienced this? What do you remember best?)

5) Heeft Laka hier een rol in gespeeld? (Did Laka play a role here?)
6) Zou (zoiets als) Laka hierin een rol kunnen spelen? (Could (something like) Laka play a role?)

*Suggesties voor verbetering (suggestions for improvement)*
Heeft u suggesties ter verbetering? Hoe zou Laka hierbij (beter) een rol kunnen spelen? (Do you have any suggestions for improvement? How could this game Laka play a (stronger) role?)

Kunt u zich nog iets herinneren over de game dat u nog niet heeft verteld? (Can you remember anything about the game that you still have not told?)
*Indien nee: Vervolg met specifieke onderwerpen. (If no: Continue with specific topics.)*

### *Specifieke onderwerpen (specific topics)*

Ik wil graag nog even een aantal onderwerpen 1 voor 1 doornemen. Laat maar weten als je we in herhaling vallen... (I'd like to go over a number of topics 1 by 1. Let me know if we fall into repetition…)

- Nieuwe thema's *(New themes)*: U vertelde dat … . Wat bedoelde u daar precies mee? (You mentioned that ... What did you mean exactly?)

- *Eerder gebruik van technologie (Previous technology usage):* Kunt u vertellen over uw eerdere ervaringen met soortgelijke systemen: internet, pc, games? (Can you tell me about your previous experience with similar systems: Internet, PC, games?)

*Gedragsfactoren (Behavioral factors)*
*Toen u Laka kreeg aangeboden… Nadat u een eerste indruk had... (When you Laka was offered ... After you had a first impression ...)*

- *Intentie (intention):* Welk voornemen of plan had u voor uzelf? Hoe zagen deze plannen eruit na enige tijd: 8 weken? (Which intention or plan did you have? How did these plans change over time: 8 weeks?)
- *(Positive) Affect:* Hoeveel zin had u erin toen u het zag? Hoe leuk? (How much sense in it you had when you saw it? How fun?)
- *Angst (anxiety):* Waren er aarzelingen om ermee aan de slag te gaan? Leg uit? (There were hesitations to get to work? Explain?)
- *Nutsverwachting (expectation of usefulness):* Wat waren (vooraf) uw verwachtingen over het nut van het spel? Welk nut heeft het daadwerkelijk gehad? (What were (before) your expectations about the usefulness of the game? What good had it actually?)
- *Verwachte moeite (expectation of effort):* Hoe dacht u vooraf over het gemak? Hoe gemakkelijk vond u het echt? Hoe kwam dat? (How do you think beforehand about convenience? How easy did you find it really? How come?)
- *Controle (control believes):* Hoe dacht u vooraf over uw kennis en kunde? Over benodigdheden? In hoeverre is het gelukt? Wat wel, wat niet? Hoe kwam dat? (How did you think in advance about your knowledge and skills? About supplies? To what extent did you succeed? What did and what did not? How come?)
- *Sociale invloed (social influence):* Met wie heeft u er vooraf over gesproken? En later? Wat dacht u over de mening van anderen? (With whom did you previously talk about it? And later? What did you think about the opinion of others?)
- *Vertrouwen (trust):* Hoe dacht u vooraf over vertrouwen, veiligheid, privacy? Heeft u hier gaandeweg nog bedenkingen over gehad? How do you think beforehand about trust, security, privacy? Did you remain having doubts about that?)
- Andere beweegredenen/gedachten? (Other motivations / thoughts?)

*Alleen indien Laka gebruikt is (*Only if Laka is used):
*Specifieke keywords spelervaring (Specific keywords game experience):*

- Uitdaging, doelen
- Feedback
- Kennis: Waar denkt u dat het over ging? maakte het nieuwsgierig, wilde u meer weten,? (Knowledge: Where do you think it was about? Were you curious, did you want to know more?)
- Controle (control)
- Concentratie, focus (concentration, attentional focus)
- Identificatie (identification)

*Suggesties ter verbetering? (Suggestions for improvement)*

***Afsluiting interview (closure)***

- Bedanken (thank you…)
- Op de hoogte houden (inform about results)
- Member check
