# Supplementary material for: Feasibility of Applied Gaming During Interdisciplinary Rehabilitation for Patients With Complex Chronic Pain and Fatigue Complaints: A Mixed-Methods Study
Source: JMIR Serious Games. 2016 Apr 1;4(1):e2. doi: 10.2196/games.5088 (PMC4833876; doi:10.2196/games.5088)
Supplement: Multimedia Appendix 7 [file games_v4i1e2_app7.pdf]

Multi-media appendix 8: [Method sensitivity analyses]

About the validity of technology acceptance scales:

- Several item distributions (concerning expectancies of performance, trust, and social influence measured at pre-adoption) revealed high kurtosis of item responses. There was a low amount of variance in these variables.
- A factor analysis was run on behavioral factor items (see Multimedia Appendix 4). An oblique rotated solution reproduced a logical structure of associations amongst constructs. Divergence between items for perceived usefulness, trust, and hedonic motivation was limited.

About missing data analysis and handling by multiple imputation:

- A Little's MCAR test (including all variables) showed that missing values on the second questionnaire amongst those who finished the first module of the game could be inferred as missing completely at random (*P*= .80).
- A frequency distribution of Mahalanobis distances (calculated over all items used to assess acceptability at t2) showed that there was no violation of the multivariate normality assumption.

About measures of association:

- Associations between ordinal and continuous variables: Spearman Rho is presented, but Kendall's tau was calculated as well → Kendall's tau is systematically smaller (as usual), but *P*-values were similar.
- Associations between dichotomous and ordinal variables: Only the Kendall's tau statistic was calculated.

About regression model robustness checks:

- Standardized residuals were inspected to determine if basic assumptions were met. Inspection of residual plots of the various models did not reveal severe violations.
- Residual analysis revealed that one case had a very strong influence on the value of R^2. A best guess is that this observation had been caused by a single measurement error. Inconsistent scale item scores of 6, 2, and 2 were observed (mean = 3.3) for the BI scale, while the predicted values for this case was 7. Deletion or value replacement produced a 5-8% improvement of explained variance across models. All observations were maintained. Multiple regression results for all participants were reproduced, or (otherwise) deleted from the results section.
- Logistic regression models were fitted for presence of positive (BI>4), higher than median (BI>5), and low (BI=<2) behavioral intentions as a method bias check for all multiple regression models. Logistic regression models including interaction effects did not include the core behavioral factors in order to limit the number of model parameters. Multiple regression results were reproduced, or (otherwise) deleted from the results section.
